# Prevalence and Antimicrobial Resistance Profiles of Foodborne Pathogens Isolated from Dairy Cattle and Poultry Manure Amended Farms in Northeastern Ohio, the United States

**DOI:** 10.3390/antibiotics10121450

**Published:** 2021-11-25

**Authors:** Woinshet Hailu, Yosra A. Helmy, Geoffrey Carney-Knisely, Michael Kauffman, Dean Fraga, Gireesh Rajashekara

**Affiliations:** 1 Center for Food Animal Health, Ohio Agricultural Research and Development Center, The Ohio State University, Wooster, OH 44691, USA; kibret.2@osu.edu (W.H.); kauffman.42@osu.edu (M.K.); 2 College of Health Sciences, Addis Ababa University, Addis Ababa, Ethiopia; 3 Global One Health initiative (GOHi), Ohio State University, Columbus, OH 43210, USA; 4 Program in Biochemistry and Molecular Biology, Department of Biology, The College of Wooster, Wooster, OH 44691, USA; geoffrey.carney-knisely@osumc.edu (G.C.-K.); dfraga@wooster.edu (D.F.)

**Keywords:** *Campylobacter*, *Salmonella*, *L. monocytogenes*, *E. coli* O157, foodborne pathogens, antimicrobial resistance, diversity, phenotypic and genotypic, correlation

## Abstract

Foodborne pathogens significantly impact public health globally. Excessive antimicrobial use plays a significant role in the development of the public health crisis of antibiotic resistance. Here, we determined the prevalence and antimicrobial resistance profiles of *E. coli* O157, *Salmonella*, *L. monocytogenes*, and *Campylobacter* isolated between 2016 and 2020 from small scale agricultural settings that were amended with dairy cattle or poultry manure in Northeastern Ohio. The total prevalence of the foodborne pathogens was 19.3%: *Campylobacter* 8%, *Listeria monocytogenes* 7.9%, *Escherichia coli* O157 1.8%, and *Salmonella* 1.5%. The prevalence was significantly higher in dairy cattle (87.7%) compared to poultry (12.2%) manure amended farms. Furthermore, the prevalence was higher in manure samples (84%) compared to soil samples (15.9%; *p* < 0.05). Multiple drug resistance was observed in 73%, 77%, 100%, and 57.3% of *E. coli* O157, *Salmonella,* *L. monocytogenes*, and *Campylobacter* isolates recovered, respectively. The most frequently observed resistance genes were *mphA, aadA*, and *aphA1* in *E. coli* O157; *blaTEM, tet(B)*, and *strA* in *Salmonella*; *penA, ampC*, *lde*, *ermB, tet(O)*, and *aadB* in *L. monocytogenes* and *blaOXA-61, tet(O)*, and *aadE* in *Campylobacter*. Our results highlight the critical need to address the dissemination of foodborne pathogens and antibiotic resistance in agricultural settings.

## 1. Introduction

Foodborne illnesses have a major public health impact in the USA and around the world. They affect approximately one in six Americans annually, leading to approximately 128,000 hospitalizations and 3000 deaths [1]. The most common foodborne pathogens include *Campylobacter*, *Salmonella*, *Escherichia coli* O157, *Listeria monocytogenes*, and *Clostridium perfringens* [2,3]. *Campylobacter, Salmonella, E. coli* O157, and *L. monocytogenes* result in about 37,000 hospitalizations and 750 deaths annually [1]. In the USA, the estimated annual cost of foodborne illness is about $90 billion per year [3,4]. For example, *Salmonella* spp. alone results in about one million infections and 400 deaths annually with a total of $4.4 billion in medical costs and lost productivity [3,5,6]. However, *L. monocytogens* infection causes the highest mortality rate compared to other foodborne pathogens with a case fatality rate of 20–30% [6] with an annual cost of about $2.6 billion [6,7]. Furthermore, *E. coli* O157:H7 causes approximately 265,000 illnesses with an estimated cost of $405 million [8,9]. *Campylobacter* infections cause 1.3 million illnesses, 13,240 hospitalizations with an estimated cost of $1.56 billion per year [5]. Foodborne pathogens cause a self-limiting gastroenteritis and do not require the use of antimicrobials, except in severe cases such as persistent enteritis, bacteremia, and in immunocompromised individuals [1,10]. However, in severe cases, antimicrobial treatments have become limited due to the rise of antimicrobial resistance among these foodborne pathogens, which complicate their treatment and have become a significant public health concern [2,11]. In the USA between 2009–2015, 5760 foodborne outbreaks were reported and the implicated bacteria were resistant to at least one antibiotic [12,13]. Out of these outbreaks, 896 were caused by *Salmonella,* 191 caused by Shiga toxin producing *E. coli* (STEC), 155 caused by *Campylobacter*, and 35 caused by *L. monocytogenes* [13]. Most of these outbreaks were associated with dairy and poultry products and vegetables [12,13]. Therefore, the USA Centers for Disease Control and Prevention (CDC) has classified drug-resistant *Campylobacter* and drug-resistant non-typhoidal *Salmonella* as serious threats to human health [2,14].

The number of antibiotic resistant bacteria isolated from humans, animals, and the environment has increased globally over the last two decades due to the overuse and misuse of antibiotics [15,16]. Currently, antibiotic resistance infections result in about 700,000 deaths worldwide. However, by 2050, if no action is taken to reduce the spread of antimicrobial resistance, the estimated number of deaths will increase by up to 10 million with more than USA $100 trillion in economic losses [17]. In the USA alone, more than 2 million infections occur due to antibiotic resistant bacteria with $20 billion in economic losses each year [17,18]. Antibiotics are often used in food animals as therapeutics or prophylactics, especially in poultry [19,20,21,22,23,24]. Additionally, several environmental components such as soil and water can act as reservoirs of antimicrobial resistance genes (ARGs) [25,26,27]. The agricultural soil may naturally contain pathogenic bacteria or receive them during soil amendment using animal manure [28]. Soil amendment with animal manure increases crop yield, but it can potentially increase the spread of foodborne pathogens and ARGs in the environment [29,30,31]. Therefore, the USA Food and Drug Administration has recently limited the use of antimicrobials on farms in the USA due to the growing impact of antibiotic resistance in clinical practice and to reduce the selection pressure on the emergence of resistance bacteria [32,33]. Here, we investigated the prevalence and phenotypic and genotypic antimicrobial resistance profiles of *E. coli* O157, *Salmonella*, *L. monocytogenes*, and *Campylobacter* isolated between 2016 and 2020 from small scale agricultural settings that were amended with dairy or poultry manure in Northeastern Ohio, USA.

## 2. Material and Methods

### 2.1. Study Area and Sample Collection

A total of 844 longitudinal manure and soil samples were collected monthly between October 2016 and October 2020 from eleven farms; five farms were amended with poultry manure, three farms were amended with dairy manure, and three farms were amended with both dairy and poultry manure. Samples were collected from a small-scale agricultural setting located in Northeastern Ohio (USA). Farms were selected based on their availability for longitudinal sampling throughout the study period and the types of manure applied (poultry or dairy). The dairy manure was obtained from open dairy heifers housed on a bedded pack during the winter and raised on pasture in the summer. The poultry manure came from boiler houses from various stages of composting. A total of 379 manure samples and 465 soil samples were collected. Six to eight fresh manure pats were collected from each farm and pooled to make one manure sample, whereas three manure samples were collected from poultry storage piles from each farm and pooled to make one manure sample. Similarly, soil samples were collected from three random sites per each field and pooled to make one soil sample. Samples were collected aseptically into Nasco Whirl-Pak™ (Fisher Scientific, Waltham, MA, USA) and stored in a cool box before transportation to the laboratory for further analyses. The numbers of the collected samples each year, their source, and the amendment type are listed in Table 1.

### 2.2. Bacterial Isolation, Enrichment, and Growth Conditions

Twenty-five grams of the samples (soil or manure) were suspended in 225 mL phosphate buffered saline (PBS; Fisher Scientific, Waltham, MA, USA) and mixed by shaking to form slurry. One mL of the slurry was then ten-fold serially diluted using PBS, and 100 µL of each serial dilution was plated onto modified charcoal cefoperazone deoxycholate agar (mCCDA; Fisher Scientific, Waltham, MA, USA), RAPID’ *L. mono* (Bio Rad, Hercules, CA, USA), sorbitol MacConkey agar plates containing cefixine and tellurite (SMACct; Becton, Dickinson; BD, San Jose, CA, USA), and xylose lysine tergitol-4 agar (XLT-4; Bio Rad, Hercules, CA, USA) plates for the direct isolation of *Campylobacter*, *L. monocytogenes*, *E. coli* O157, and *Salmonella*, respectively. Samples that did not show any bacterial growth due to direct plating were subjected to enrichment using pathogen specific media [34,35,36,37,38].

The enrichment and isolation of *E. coli* O157 was performed as previously described [34]. Briefly, 10 mL of the slurry was enriched in 90 mL of buffered peptone water (BPW; Thermo Scientific, Waltham, MA, USA) and incubated at 42 °C for 18–24 h. Automated immunomagnetic separation (AIMS) was performed on BPW enriched samples using anti-O157 specific immunomagnetic beads (Invitrogen, Waltham, MA, USA). The recovered beads were plated on SMACct and incubated at 37 °C for 18–24 h. An *E. coli* O157 Latex test (Oxoid Ltd., Cambridge, UK) was used to confirm the presence of *E. coli* O157. The isolated colonies were preserved at −80 °C in brain heart infusion broth (BHI; BD Difco, Franklin Lakes, NJ, USA) containing 30% glycerol (*v*/*v*) for further analysis.

To isolate *L. monocytogenes*, 10 mL of the slurry was added to 90 mL universal pre-enrichment broth (UPB; Oxoid Ltd., Cambridge, UK) and incubated at 35 °C for 24 h. One mL of the enriched culture was transferred to nine mL of Fraser broth (Oxoid Ltd., Cambridge, UK) and incubated at 35 °C for 24 h. A loopful (10 µL) of darkened Fraser broth was streaked on PALCAM plates (Neogen, Lansing, MI, USA) and incubated at 35 °C for 48 h. The grown colonies were subcultured on Rapid’ *L. mono* plate (Neogen, Lansing, MI, USA) and incubated at 37 °C for 24 h. Blue colonies without a yellow halo were collected and preserved at −80 °C in BHI broth containing 30% glycerol (*v*/*v*) for further analysis [35,36].

For *Salmonella* isolation, 10 mL of the slurry was enriched in 90 mL tetrathionate broth (Oxoid Ltd., Cambridge, UK) and incubated at 37 °C for 24 h. One mL of the grown culture was then transferred to ten mL of Rappaport-Vassiliadis broth (RV; Oxoid Ltd., Cambridge, UK) and incubated at 37 °C for 24 h. A loopful of bacteria grown in RV were plated on XLT-4 plates and incubated at 37 °C for 24 h [37]. Black suspect colonies were preserved at −80 °C in BHI broth containing 30% glycerol (*v*/*v*) for further analysis.

To isolate *Campylobacter* spp., 1 mL of manure or soil slurry was suspended in 9 mL of Preston enrichment broth containing *Campylobacter* growth supplements (CM067, SR048, SR117, and SR232; Oxoid Ltd., Cambridge, UK) [38]. The suspensions were incubated under microaerobic conditions (5% O_2_, 10% CO_2_, and 85% N_2_) at 42 °C for 48 h. After enrichment, 100 µL was plated on mCCDA containing a selective supplement (SR0155; Oxoid Ltd., Cambridge, UK) and incubated under microaerobic conditions at 42 °C for 48 h. The isolated colonies from mCCDA plate were then subcultured onto Mueller–Hinton (MH) agar containing a selective supplement (SR0117; Oxoid Ltd., Cambridge, UK) and incubated at 42 °C for 48 h under microaerobic conditions [39,40,41]. The grown *Campylobacter* cultures were frozen at −80 °C in MH broth supplemented with 30% glycerol (*v*/*v*) until further use.

### 2.3. DNA Extraction and PCR Analysis for Bacterial Species Identification

Bacterial DNA was extracted using the boiling method [39]. Briefly, a half loop from bacterial cultures were suspended in 100 μL of sterile DNase-free water, heated at 95 °C for 10 min, cooled, and centrifuged at 4000× *g* for 10 min. The supernatants containing the nucleic acids were collected in new tubes and stored at −20 °C. In cases where no PCR products were detected, template DNA was prepared using a MasterPure™ Complete DNA and RNA Purification Kit (Epicenter, Madison, WI, USA) according to the manufacturer’s instructions. Confirmation of *bacterial* spp. for *E. coli* O157, *Salmonella L. monocytogenes*, and *Campylobacter* was performed using a multiplex-PCR assay (mPCR) [39,42,43,44]. PCR products were visualized using gel-electrophoresis on a 2% agarose gel containing 0.5 µg/mL ethidium bromide. The mPCR conditions, target gene, primers sequence, and amplicon size are listed in Table S1.

### 2.4. Antimicrobial Susceptibility Testing

Antimicrobial susceptibility testing was carried out using the broth microdilution method [45]. *E. coli* O157 and *Salmonella* isolates were tested for their susceptibility to aminoglycosides: kanamycin (Kan), streptomycin (Str), and gentamicin (Gen); β–lactam combination agents: amoxicillin-clavulanic acid (Amo); cephems: cefoxitin (Cefo) and ceftriaxone (Ceft); folate pathway antagonists: sulfisoxazole (Sul) and trimethoprim- sulfamethoxazole (Tri); macrolides: azithromycin (Azi); penems: meropenem (Mer); penicillin: ampicillin (Amp); phenicols: chloramphenicol (Chl); quinolones: ciprofloxacin (Cip), nalidixic acid (Nal); tetracyclines: tetracycline (Tet); and polymyxins: colistin (Col) (Sigma-Aldrich, St. Louis, MO, USA). *L. monocytogenes* isolates were screened for susceptibility to aminoglycosides: kanamycin (Kan), streptomycin (Str), and gentamicin (Gen); cephems: cefoxitin (Cefo) and ceftriaxone (Ceft); folate pathway antagonists: trimethoprim- sulfamethoxazole (Tri); macrolides: azithromycin (Azi), erythromycin (Ery); penems: meropenem (Mer); penicillin: ampicillin (Amp) and penicillin G (Pen); rifamycins: rifampicin (Rif); phenicols: chloramphenicol (Chl); quinolones: ciprofloxacin (Cip); nalidixic acid (Nal); tetracyclines: tetracycline (Tet); nitrofuran: nitrofurantoin (Nit); oxazolidinone: linezolid (Lin); fluoroquinolone: levofloxacin (Lev); lincomycin: clindamycin (Clin); and glycopeptide: vancomycin (Van). *Campylobacter* isolates were screened for susceptibility to aminoglycosides: kanamycin (Kan), streptomycin (Str), and gentamicin (Gen); macrolides: azithromycin (Azi) and erythromycin (Ery); penicillin: ampicillin (Amp) and penicillin G (Pen); phenicols: chloramphenicol (Chl); quinolones: ciprofloxacin (Cip), nalidixic acid (Nal); tetracyclines: tetracycline (Tet); lincomycin: clindamycin (Cli); ketolides: telithromycin (Tel); and amphenicol: florfenicol (Flo). These antimicrobials are representatives of the drugs used for humans and in the animal industry and were chosen according to the National Antimicrobial Resistance Monitoring System (NARMS) records [46].

Briefly, *E. coli* O157 and *Salmonella* cultures were suspended in LB (Luria-Bertani) broth while *L. monocytogenes* cultures were suspended in UPB broth and *Campylobacter* cultures were suspended in MH broth to achieve an optical density (OD_600_) of 0.05. One hundred microliters of a suspension were added to each well of the 96-well plate containing two-fold serial dilutions of the antimicrobial agents. Positive and negative control wells contained bacterial suspensions without antimicrobials, and sterile broth containing each of the antimicrobials, respectively. The *E. coli* O157 and *Salmonella* plates were then incubated under aerobic conditions at 37 °C for 24 h [47], while the plates for *L. monocytogenes* were incubated for 48 h under aerobic condition at 37 °C and the *Campylobacter* plates were incubated at 42 °C under microaerobic condition [47,48,49]. The plates were assessed visually and using a spectrophotometer (Tecan Group Ltd., San Jose, CA, USA) to determine growth inhibition. Minimum inhibitory concentration (MIC) values were defined as the lowest concentration of an antimicrobial agent that produced no visible growth. The isolates that possessed resistance to three or more classes of antimicrobials were considered multi-drug resistant (MDR) [50]. The test was performed in accordance with the recommendations of the Clinical Laboratory Standards Institute (CLSI) criteria by using the available CLSI breakpoint interpretive criteria (Table S2).

### 2.5. Detection of Antibiotic Resistance Genes (ARGs)

The most prevalent ARGs for each antibiotic were selected and screened for their presence in the foodborne pathogens. *E. coli* O157 isolates were screened for six ARGs: tetracycline resistant gene *tet(A)*, aminoglycoside acetyltransferase gene *aac (3)-IV*, streptomycin adenyl transferase gene *aadA*, aminoglycoside 3′-phosphotransferase gene *aphA1*, sulfonamide resistant gene *sulII*, and macrolide resistance gene *mphA*. *Salmonella* isolates were screened for six ARGs: beta-lactamase gene *bla _TEM_,* streptomycin resistant gene *strA*, tetracycline resistant gene *tet (B)*, aminoglycoside acetyltransferase gene *aac (3)-Iva*, sulfonamide resistant gene *sulII*, and macrolide resistant gene *mphA*. *L. monocytogenes* isolates were screened for nine ARGs: ampicillin resistant gene *ampC,* ciprofloxacin resistant gene *lde,* erythromycin resistant gene *ermB,* tetracycline resistant gene *tet(O),* gentamicin resistant gene *aadB,* penicillin G resistant gene *penA,* cefoxitin resistance gene *cfxA,* macrolide resistant gene *mefA*, and sulfonamide resistant gene *sulI. Campylobacter* isolates were screened for ampicillin resistant gene *blaOXA-61*, streptomycin resistant gene *aadE,* tetracycline resistant gene *tet(O)*, and gentamycin resistant gene *aph-3-1*. Amplification was performed in a 25 µL reaction volume containing 12.5 µL of GoTaq Green Master Mix (2X; 2.5 units) (Promega, Madison, WI, USA), 3 µL of template DNA, and 0.2 µM of each forward and reverse primer. The PCR was performed with 3 min of initial denaturation at 95 °C, followed by 35 cycles of denaturation at 95 °C for 1 min, annealing at optimal annealing temperature for 45 s, extension at 72 °C for 1 min, and final extension at 72 °C for 5 min. Primer sequence, expected amplicon size, and annealing temperatures are described in Table S3. Nuclease-free water was used as a negative control. PCR products were visualized on a 2% agarose gel containing 0.5 µg/mL ethidium bromide under UV light.

### 2.6. Statistical Analysis

Statistical analysis was performed in IBM SPSS 26.0 using one-way analysis of variance (ANOVA) followed by Tukey’s posttest. Student’s *t*-test and the chi-square test were used for pairwise comparisons of differences in the resistance rates for each antimicrobial agent between poultry and dairy manure amended farms. A *p*-value of <0.05 was considered statistically significant difference. Linear regression analysis was used for the trend analysis of the prevalence and antimicrobial resistance during the study period. JMP Pro 15 was used to plot heatmap representation with dendrogram of each foodborne pathogen. Principal component analysis (PCA) was used to visualize the distribution of phenotypic and genotypic resistance. The statistical analysis of the correlation between phenotypic and genotypic resistance was performed in the vegan package on R studio (SAS institute Inc., Cary, NC, USA).

## 3. Results

### 3.1. Prevalence and Distribution of Campylobacter, E. coli O157, Salmonella, and L. monocytogenes

**Figure 1:**
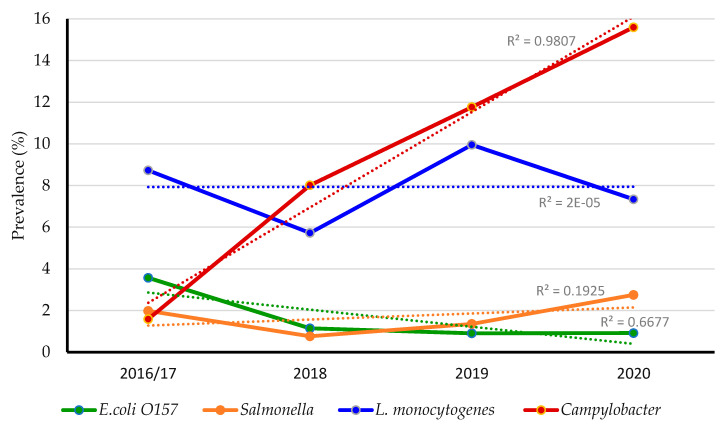
Prevalence of foodborne pathogens in dairy cattle and poultry manure amended farms between 2016 and 2020.

A total of 19.3% (163 of 844) of the collected samples were positive for at least one of the foodborne pathogens. Out of these, 82.2% (134 of 163) of samples had a single pathogen, while 17.8% (29 of 163) of samples had more than one pathogen. Notably, the total prevalence of *Campylobacter* (8%; 68 of 844) and *L. monocytogenes* (7.9%; 67 of 844) was higher than *E. coli* O157 (1.8%; 15 of 844) and *Salmonella* (1.5%; 13 of 844). However, 93.1% (27 of 29 isolates) had contamination with different pathogens; *L. monocytogenes + Campylobacter* (44.8%; 13 of 29 isolates)*, Campylobacter + Salmonella* (13.7%; 4 of 29 isolates)*, Campylobacter + E. coli* O157 (10.3%; 3 of 29 isolates)*, L. monocytogenes + Salmonella* (6.8%; 2 of 29 isolates), *L. monocytogenes + E. coli* O157 (13.7%; 4 of 29 isolates), *E. coli* O157 *+ Salmonella* (3.4%; 1 of 29 isolates), and *Salmonella + L. monocytogenes + Campylobacter* (6.8%; 2 of 29 isolates). None of the samples were positive for all four tested foodborne pathogens. Notably, the prevalence of the foodborne pathogens had not increased from 2016 to 2020 except for *Campylobacter*, which showed significant increase throughout the study years (R of 0.9; *p* < 0.05; Figure 1). This increase might be attributed to climate changes, rainfall, and soil properties [51,52,53]. Moreover, out of 67 *L. monocytogenes* isolates recovered, 5.9% (4 of 67) were 1/2a serotype, 26.8% (18 of 67) were 1/2b serotype, 19.4% (13 of 67) were 4b serotype, and the remaining 47.7% (32 of 67) were untypable.

Notably, the prevalence of the foodborne pathogens was significantly higher in dairy cattle (87.7%) compared to poultry manure amended farms (12.3%; *p* < 0.05; Table 2). In dairy cattle manure amended farms, *Campylobacter* (14.9%) was the most prevalent compared to *L. monocytogenes* (13.1%), *E. coli* O157 (2.9%), and *Salmonella* (1.4%), whereas in poultry manure amended farms, *L. monocytogenes* (2.2%) was the most prevalent compared to *Salmonella* (1.7%) and *Campylobacter* and *E. coli* O157 (0.5%). The prevalence of all pathogens was higher in manure samples (84%) compared to soil samples (15.9%; *p* < 0.05; Table 2). In manure samples, *Campylobacter* (17.7%) was the most prevalent than *L. monocytogenes* (12.4%), *Salmonella* (3.2%), and *E. coli* O157 (2.9%), whereas in soil samples, *L. monocytogenes* (4.3%) was the most observed foodborne pathogen followed by *E. coli* O157 (0.9%), *Salmonella*, and *Campylobacter* (0.2%; Table 2).

### 3.2. Antimicrobial Resistance Phenotypical Profile of E. coli O157, Salmonella, L. monocytogenes and Campylobacter

All isolates that were confirmed positive for *E. coli* O157, *Salmonella*, *L. monocytogenes*, and *Campylobacter* were used for broth microdilution assay. Collectively, all the tested isolates possessed resistance to at least one of the tested antimicrobials. *E. coli* O157, *Salmonella*, and *L. monocytogenes* isolates possessed 100% resistance to Tri. Additionally, *E. coli* O157 and *Salmonella* isolates showed 100% resistance to Sul, whereas *Campylobacter* isolates possessed 100% resistance to Pen. Interestingly, most of the *E. coli* O157 isolates were susceptible to the tested antibiotics, however, most of the *L. monocytogenes* isolates were resistant to the tested antibiotics. For example, *E. coli* O157 isolates possessed 100% susceptibility to Chl, Cip, Nal, Ceft, and Mer, while *L. monocytogenes* isolates possessed 100% resistant to Ceft, Cefo, Cli, Rif, Tri, Mer, and Azi. The antimicrobial resistance profile of different foodborne pathogens is shown in Table 3.

Our results also showed that *E. coli* O157 isolates from poultry manure amended farms were 100% resistant to Amp, Str, Gen, Sul, and Tri, whereas in dairy cattle manure amended farms, 100% of *E. coli* O157 isolates were resistant to Sul and Tri (Table S4). Interestingly, 73% (11 of 15) of the *E. coli* O157 isolates revealed MDR to aminoglycosides (100%), macrolides (73%; 11 of 15), and folate antagonists (100%). Further, *Salmonella* isolates recovered from poultry manure amended farms possessed 100% resistant to Gen, Sul, Tri, and Azi, while isolates recovered from dairy cattle manure amended farms were 100% resistant to Sul and Tri (Table S5). A total of 77% (10 of 13) of the *Salmonella* isolates possessed MDR to aminoglycosides (84%; 11 of 13), macrolides (77%; 10 of 13), and folate antagonists (100%). Notably, all *L. monocytogenes* isolates recovered from poultry manure amended farms were resistant to Kan, Nal, and Lev. However, more than 50% of the *L. monocytogenes* isolates recovered from dairy cattle manure amended farms were resistant to Kan, Nal, and Lev (Table S6). We also found that all *L. monocytogenes* isolates showed MDR to cephems, lincomycin, antimycobacterial, folate antagonist, penem, and macrolides with 100% resistance for each antimicrobial class. Additionally, *Campylobacter* isolates recovered from poultry manure amended farms were 100% resistant to Amp, Nal, and Pen, while isolates recovered from dairy cattle manure amended farms were 100% resistant to Ery and Pen (Table S7). A total of 57.3% (39 of 68) of the *Campylobacter* isolates showed MDR to aminoglycosides (54%; 37 of 68), tetracyclines (63%; 43 of 68), and penicillin (100%). The resistance of the foodborne pathogens to the tested antimicrobials did not increase throughout the study period from 2016 to 2020 (Figure S1). On the other hand, manure samples contained more resistant isolates compared to soil samples. Details about the antimicrobial profile of the *E. coli* O157, *Salmonella, L. monocytogenes*, and *Campylobacter* isolates from manure and soil samples collected from dairy cattle and poultry manure amended farms are shown in Tables S4–S7.

### 3.3. Genotypic Profile of Antimicrobial Resistance in E. coli O157, Salmonella L. monocytogenes and Campylobacter Isolates

Phenotypically antimicrobial resistant isolates of different foodborne pathogens were tested for the presence of the corresponding ARGs. Regardless of the type of the isolated pathogen, more ARGs were detected in dairy cattle than poultry manure amended farms. Our results showed that the *E. coli* O157 isolates contained 100% of *aphA1* and *mph A* genes. Further, *aadA*, *tetA*, *aac (3)-IV*, and *Sul II* genes were detected in 75% (6/8), 50% (1/2), 84% (11/13), and 63% (7/15) of the *E. coli* O157 isolates, respectively. Notably, none of the *Salmonella* isolates contained the Azi resistant *ermB* gene. However, 66% (4/6), 66% (2/3), 63% (7/11), 40% (4/10), and 15% (2/13) of *Salmonella* isolates contained *blaTEM*, *tetB*, *strA*, *aac (3)-Iva,* and *sul II* genes, respectively (Figure S2).

None of the *L. monocytogenes* isolates contained the cefoxitin resistance gene *cfxA*, macrolide resistance gene *mefA*, and sulfonamide resistance gene *sulI.* Regardless of the source of the isolates, the most frequently detected ARGs within *L. monocytogenes* isolates were *lde* 86.7% (46/53) followed by *ampC* 66% (40/60), *aadB* 51.9% (27/52), *penA* 50% (16/32), *ermB* 28% (7/25), and *tet(O)* 8% (2/23). However, the *penA* gene was detected only in dairy cattle manure amended farms. Notably, none of the *Campylobacter* isolates contained the gentamycin resistance *aph-3-1* gene. However, *Campylobacter* isolates contained 90% (57/63), 79% (34/43), and 73% (11/15) of *blaOXA-61*, *tet(O)*, and *aadE* genes, respectively. The prevalence of ARGs was higher in the foodborne pathogens isolated from dairy cattle compared to poultry manure amended farms. Regardless of the type of pathogen, 88% (260 of 304) of the resistance genes were detected in dairy manure amended farms, while 11% (36 of 304) were detected in poultry manure amended farms (*p* < 0.05). The prevalence of ARGs in *E. coli* O157, *Salmonella*, *L. monocytogenes*, and *Campylobacter* isolates recovered from different sources is shown in Figure S2.

### 3.4. Correlation between Phenotypic and Genotypic Resistance of the Foodborne Pathogens

**Figure 2:**
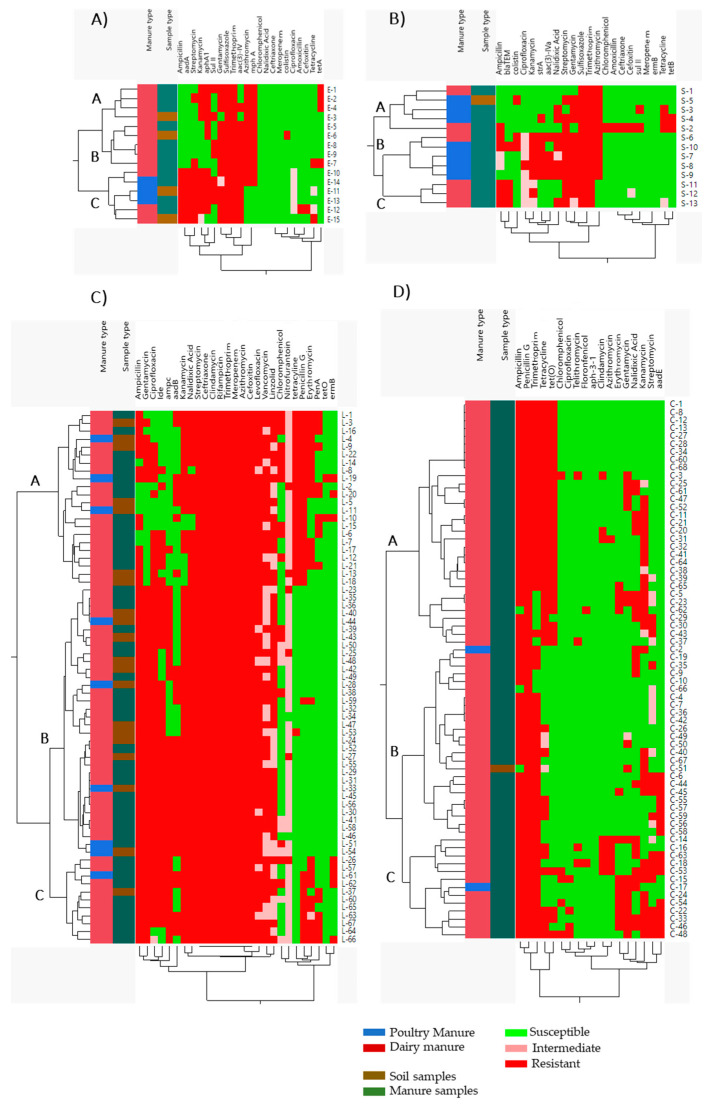
Hierarchical clustering of (**A**) *E. coli* O157, *(***B**) *Salmonella*, (**C**) *L. monocytogenes,* and (**D**) *Campylobacter.* Sample clustered according to their manure amendment type and phenotypic and genotypic antibiotic resistance profile. Red color, phenotypic resistance and presence of ARGs; green color, susceptible and absence of ARGs; blue color, poultry manure amendment; dark red color, dairy manure amendment; dark green color, manure samples; and brown color, soil samples.

Hierarchical clustering, correlation matrix analysis, and PCA were used to determine the associations between the phenotypic and genotypic characteristics and the source of the isolates. The hierarchical clustering showed that four *E. coli* O157 isolates that possessed resistance to Kan, Gen, Sul, Tri, and Azi, susceptibility to Chl, Nal, Cef, Mer, Col, Cip, Amo, Ceft, and Tet, and contained *aphA1* and *mphA* genes were clustered together (Cluster A). Further, another five isolates were clustered together and they showed resistance to Gen, Sul, Tri, and Azi, susceptibility to Amp, Chl, Nal, Ceft, Mer, Cip, Amo, and Ceft, and contained *aac(3)-IV* and *mphA* genes (Cluster B). Cluster A and B isolates originated from dairy manure amended farms. On the other hand, six of the *E. coli* O157 isolates were clustered together and showed resistance to Amp, Str, Sul, and Tri, susceptibility to Chl, Nal, Ceft, Mer, and Col, and contained *aadA* and *aac(3)-IV* genes (Cluster C), however, they originated from both poultry and dairy cattle manure amended farms (Figure 2A). Similarly, five isolates of *Salmonella* were clustered together and showed resistance to Azi and susceptibility to Cip, Kan, and Mer (Cluster A; Figure 2B). In cluster B, five isolates possessed resistance to Kan, Str, Gen, Sul, Tri, and Azi, susceptibility to Chl, Amo, Ceft, Cefo, Mer, and Tet, and contained the *strA* gene, while in cluster C, three isolates that showed resistance to Amp, Sul, and Tri, susceptibility to Chl, Amo, Ceft, and Mer, and contained the *blaTEM* gene were clustered together. Cluster A and B isolates originated from both poultry and dairy manure amended farms, whereas in cluster C isolates originated only from dairy manure amended farms (Figure 2B).

Notably, 22 of *L. monocytogenes* isolates were clustered together and possessed resistance to Ceft, Cli, Rif, Tri, Mer, Azi, Cefo, Tet, and Pen (Cluster A; Figure 2C). However, in cluster B, 34 isolates were resistant to Amp, Gen, Ceft, Cli, Rif, Tri, Mer, Azi, Cefo, and Cip, and in cluster C, eleven isolates that were resistant to Amp and Gen, susceptible to Tet, and contained the *ampC* gene were clustered together. Isolates in cluster A and B contained variable ARGs. Cluster A, B, and C isolates originated from both poultry and dairy manure amended farms (Figure 2C). Similarly, 32 *Campylobacter* isolates that were resistant to Pen, Tet, and possessed the *tet(O)* gene (only one isolate did not have *tet(O)*) were clustered together (Cluster A; Figure 2D). Within this cluster, eight isolates were phenotypically and genotypically similar. All isolates in this cluster originated from dairy cattle manure amended farms. However, in cluster B, 23 isolates were resistant to Pen and susceptible to Chl, Cip, Tel, and Flo, whereas in cluster C, 13 isolates resistant to Amp and Pen were clustered together. Cluster B and C isolates originated from both poultry and dairy manure amended farms (Figure 2D).

**Figure 3:**
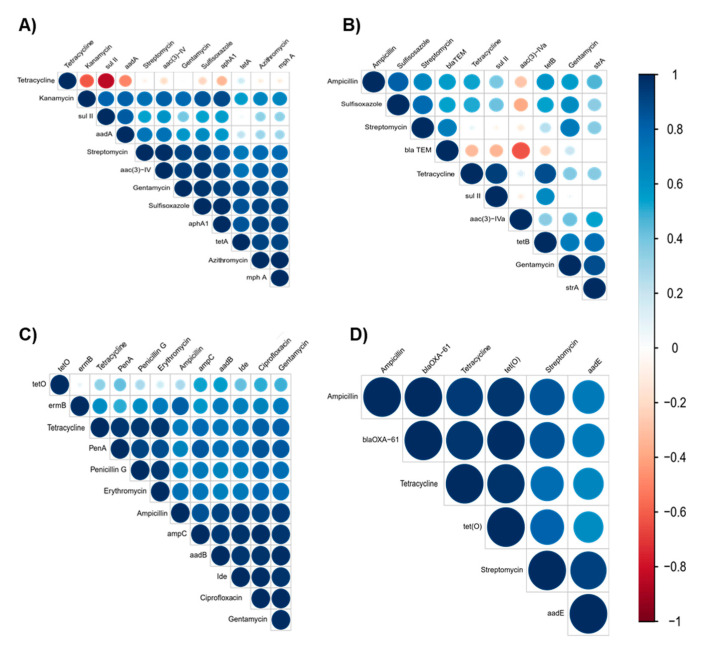
Correlation matrix of phenotypic and genotypic antibiotic resistance of (**A**) *E. coli* O157, (**B**) *Salmonella*, (**C**) *L. monocytogenes,* and (**D**) *Campylobacter.* Blue is positive correlation and red is negative correlation (1 = positive correlation, 0 = no correlation, and −1 = negative correlation). Size and strength of the color represent numerical value of correlation coefficient.

The correlation analysis revealed that the tested ARGs were significantly positively correlated to their corresponding antimicrobials (*p* < 0.05). There was a strong positive correlation between Azi and *mph A* (*r* = 0.8), Kan and *aphA* (*r* = 0.8), Str and *aadA* (*r* = 0.7), and Gen and *aac 3 IV* (*r* = 0.9) in *E. coli* O157 isolates (Figure 3A), whereas in *Salmonella* isolates, Tet and *tet B* were positively corelated (*r* = 0.9; *p* < 0.05; Figure 3B). Similarly, in *L. monocytogenes* isolates, there were strong positive correlations between Cip and *Ide* (*r* = 0.9), Pen and *pen A* (*r* = 0.9), Ery and *ermB* (*r* = 0.7), Amp and *ampC* (*r* = 0.8), and Gen and *aadB* (*r* = 0.9) (*p* < 0.05; Figure 3C). In *Campylobacter* isolates, Tet and *tet*O (*r* = 0.9) and Amp and *bla*OX-61 (*r* = 0.9) showed significant positive correlation (Figure 3D). This indicates that the detected resistance genes were the determinants for the observed phenotypic resistance in these pathogens [54]. We also observed a strong positive correlation between ARGs and their non-corresponding antimicrobials (*p* < 0.05). For example, there were positive correlations between Kan and *aac 3 IV* (*r* = 0.8) and Str and *aac 3 IV* (*r* = 0.9) in *E. coli* O157 isolates and Gen and *strA* (*r* = 0.8) in *Salmonella* isolates. The observed correlation might be due to the action of the ARGs to antimicrobials that belong to the same class [55]. Additionally, there was a positive correlation between ARGs and antimicrobials from different classes. For example, Gen and *mphA* (*r* = 0.8) in *E. coli* O157, Gen and *TetB* (*r* = 0.7) in *Salmonella* isolates, Ery and *penA* (*r* = 0.8), Gen and *penA* (*r* = 0.8), Amp and *ermB* (*r* = 0.8), and Ery and *Ide* (*r* = 0.7) in *L. monocytogenes* isolates, and Amp and *aadE* (*r* = 0.7) and Gen and *aadE* (*r* = 0.5) in *Campylobacter* isolates were positively correlated (*p* < 0.05; Figure 3). We also found a strong correlation between different antibiotics. For instance, a strong positive correlation was observed between Sul and Azi (*r* = 0.9), Str and Gen (*r* = 0.8), and Str and Sul (*r* = 0.9) in *E. coli* O157 isolates, Gen and Sul (*r* = 0.7) and Gen and Str (0.7) in *Salmonella* isolates, Cip and Gen (*r* = 0.9) and Pen and Tet (*r* = 0.9) in *L. monocytogenes* isolates, and Amp and Str (*r* = 0.8) and Tet and Amp (*r* = 0.9) in *Campylobacter* isolates (Figure 3). This observed strong correlation could be associated with the occurrence of MDR in these isolates [56]. These results suggest that manure amended farms that contain resistance pathogens could be important hotspots for the spread of antimicrobial resistance and ARGs between isolates and can cause a significant public health risk [57,58,59].

**Figure 4:**
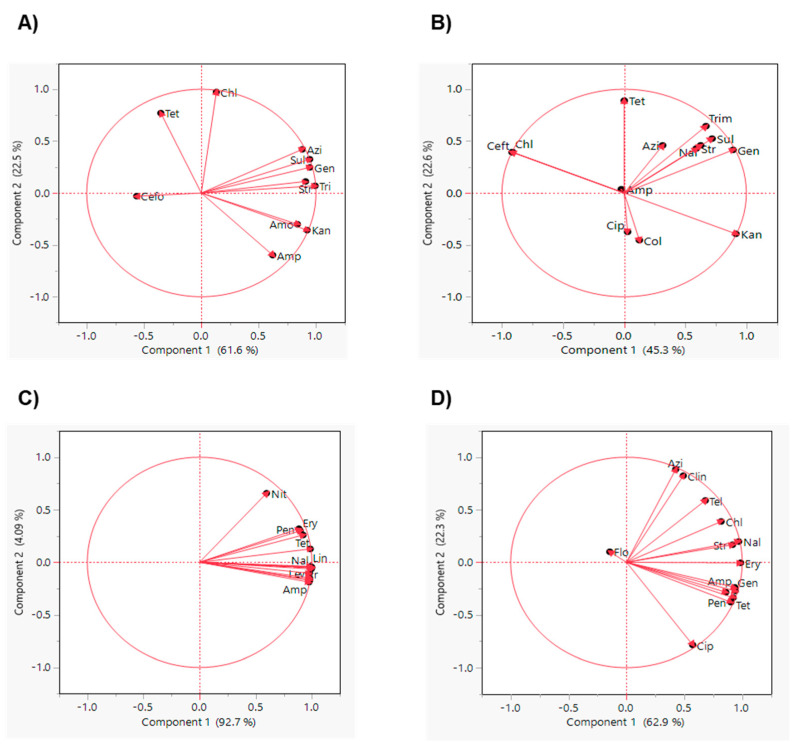
Principal component analysis of relative abundance of phenotypic antimicrobial resistance of (**A**) *E. coli* O157, (**B**) *Salmonella*, (**C**) *L. monocytogenes,* and (**D**) *Campylobacter.* Amp: ampicillin, Chl: chloramphenicol, Cip: ciprofloxacin, Kan: kanamycin, Nal: nalidixic acid, Str: streptomycin, Tet: tetracycline, Gen: gentamycin, Sul: sulfisoxazole, Ceft: ceftriaxone, Amo: amoxicillin, Cefo: cefoxitin, Azi: azithromycin, Tri: trimethoprim sulfamethoxazole, Mer: meropenem, Col: colistin, Pen: penicillin G, Ery: erythromycin, Van: vancomycin, Lin: linezolid, Nit: nitrofurantoin, Cli: clindamycin, Lev: levofloxacin, Tel: telithromycin, Flo: florfenicol.

The PCA analysis showed that Sul, Tri, Gen, Str, and Azi resistant isolates and Amo and Kan resistant isolates of *E. coli* O157 were grouped together, showing their similar trend of occurrence within these isolates. However, Tet, Cefo, and Chl stand separately as they do not have a similar trend of occurrence to other antibiotics (Figure 4A). In *Salmonella*, Sul, Tri, Gen, Nal, and Str resistant isolates were grouped together, emphasizing their similar trends of occurrence within these isolates (Figure 4B). Similarly, Chl and Ceft and Cip and Col were close to each other, but the Tet resistant isolates stood separately from these antimicrobials (Figure 4B). These PCA results were supported by the correlation analysis where Sul, Gen, and Str strongly corelated in both *E. coli* O157 and *Salmonella* isolates (Figure 3A,B). On the other hand, except for Nit, the other antibiotics were grouped together in *L. monocytogenes* isolates (Figure 4C), suggesting that Nit does not have a similar trend of occurrence to the other antibiotics. A similar result was also shown in the hierarchical clustering (Figure 2C). Additionally, Ery, Amp, Gen, Tet, and Pen resistant *Campylobacter* isolates were close to each other (Figure 4D), explaining their similar trend of occurrence and strong correlation (Figure 3D). Furthermore, Nal and Str and Azi and Cli resistant isolates were also grouped together, while Cip resistant isolates were far from the other antibiotics and possessed a different occurrence trend compared to the other antibiotics (Figure 3D).

**Figure 5:**
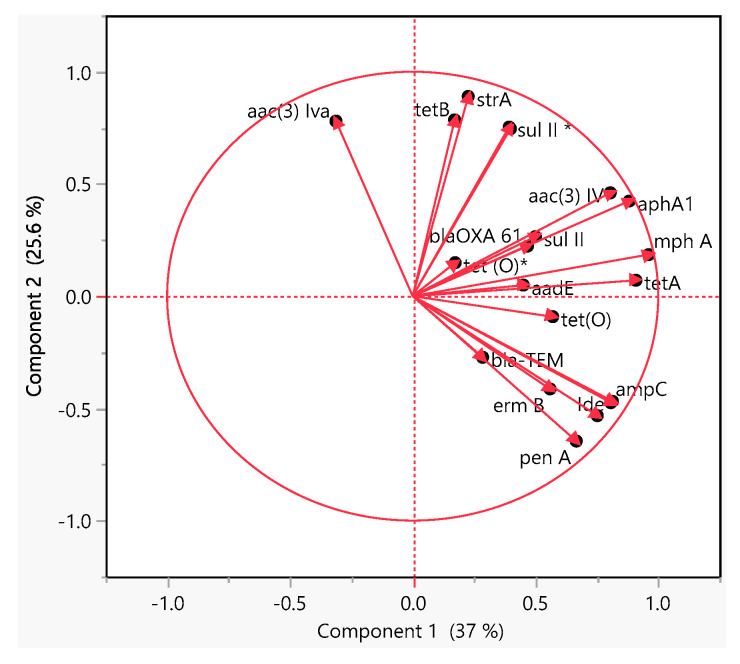
Principal component analysis of the abundance of ARGs in all foodborne pathogens: for *E. coli* O157, *tet(A)*, *aac(3)-IV*, *aphA1*, *sulII, aadA*, and *mphA* genes; for *Salmonella*, *strA*, *tet (B)*, sulII*, blaTEM, and *aac(3)-Iva*; for *L. monocytogenes*, ampC, lde, ermB, tet(O)*, aadB, and *penA*; and for *Campylobacter*, *blaOXA-61*, *aadE,* and *tet(O)*.

The PCA analysis of the ARGs showed that *mphA* and *tetA* and *aac (3)-IV* and *aphA1* in *E. coli* O157 isolates were grouped together (Figure 5), indicating their similar trend of occurrence as shown in the hierarchical clustering (Figure 2A). However, *tetB* and *strA* genes in *Salmonella* isolates were grouped together (Figure 5) and have strong correlation (Figure 4B). Additionally, *penA, Ide* and *ampC* in *L. monocytogenes* isolates and *blaOXA-61* and *tet(O)* in *Campylobacter* isolates (Figure 5) showed similar occurrence and they clustered together. Notably, *L. monocytogenes* isolates possessed strong positive correlation (Figure 3C; *p* < 0.05), while *Campylobacter* isolates clustered together (Figure 2D).

## 4. Discussion

The widespread use and misuse of antibiotics in food animal production systems has resulted in the emergence of antibiotic resistant zoonotic bacteria that can be transmitted to humans through the food chain [60]. Infection with antibiotic resistant bacteria negatively impacts the public health due to an increased incidence of treatment failure and severity of disease [61].

Interestingly, the total prevalence of foodborne pathogens in this study (19.3%) is lower than in previous reports [62,63,64]. The prevalence of *E. coli* O157 (1.8%) and *Salmonella* (1.5%) (Table 2) is lower than in previous reports conducted in California [65,66,67]. The total prevalence of *L. monocytogenes* (7.9%; Table 2) is lower than in previous reports in Ohio, New York and other states of the US [68,69,70]. Furthermore, the prevalence of *Campylobacter* (8%; Table 2) is lower than the detected prevalence in Michigan [71]. In our study, *E. coli* O157 was detected only in 2.9% of dairy cattle manure amended farms. The previously detected prevalence of *E. coli* O157 in cattle in Ohio increased from 2.1% in 2002 [72] to 24% in 2009 [73]. However, at the global level, the estimated prevalence of *E. coli* O157 in cattle ranged between 0.13% and 61.8% [74]. The prevalence of *Salmonella* (1.4%) in dairy cattle manure is lower than in previous reports from Texas [75]. Additionally, the detected prevalence of *Salmonella* in poultry manure amended farms (1.7%) is lower than the detected prevalence in Georgia (6%) and North Carolina (26%) [76,77]. *L. monocytogenes* prevalence in poultry (2.2%) and dairy cattle (13.1%) manure amended farms (Table 2) is lower than the detected prevalence in 2010 in Ohio, New York, and southeastern US [78,79,80]. The prevalence of *Campylobacter* among dairy cattle manure amended farms in our study (14.9%; Table 2) is higher than the findings reported in dairy cattle feces in other localities (northeastern, north, east, midwest, and south) in the US [68,71,81]. Interestingly, the prevalence of *Campylobacter* in poultry manure amended farms (0.5%) in northeastern Ohio (Table 2) is lower than the detected prevalence (69%) in northwestern Ohio [82] and Maryland [83]. Our result revealed that 1/2a serotype of *L. monocytogenes* isolates is less prevalent (5.9%) than similar studies previously conducted in Ohio and other states of the US [67,69,84]. Regardless of the detected bacteria, the variation in the prevalence estimates might be due to differences in the husbandry and management systems, type of feed, geographical locations, and the concentration of the farms in each location [85,86], which can have an impact on the dissemination and transmission of pathogens. Additionally, in poultry this variation might also be due to seasonal effects and differences in the hatchery sources, feed composition, vaccination programs, and flock-disease status [77]. Contamination of agricultural farms with different foodborne pathogens might be attributed to using either dairy or poultry manure amendments [87]. Other factors might contribute to the contamination of agricultural farms by pathogens including weather parameters, rainfall, and soil properties (hydraulic properties, texture, soil cover slope), however, these factors were not studied in our study [51,52,53].

Antimicrobial resistance in foodborne pathogens isolated from different sources appears to be increasing in many countries. Previous studies have indicated that both poultry and dairy cattle manure could be sources of antibiotic resistance bacteria [63,88,89]. In our study, 73%, 77%, 100%, and 57.3% of *E. coli* O157, *Salmonella*, *L. monocytogenes*, and *Campylobacter* isolates were resistant to at least one of the tested antimicrobial classes (Table 3), respectively. These results were higher than previous studies conducted on the same pathogens in Ohio and other states of the US [69,90,91,92]. The relatively high percentages of resistance to the tested antimicrobials might be due to differences in antimicrobial agents use [93]. The antimicrobial resistance in *E. coli* O157 isolates to aminoglycoside (Str) is higher among dairy cattle farms than poultry farms. A previous study showed that *E. coli* O157:H7 isolates from food animals have a higher rate of resistance to Amp, Str, Kan, and Tet [94]. The usage of these classes of antimicrobials has increased more than 20% between 2009 and 2016 [95]. The resistance of *E. coli* O157 isolates to Gen (84.6%) in dairy cattle feces (Table S4) is significantly higher than the previously reported prevalence in Ohio and the west, midwest, northeast, and southeast states of the US [96,97]. Additionally, the prevalence of Gen and Tet resistance in *Salmonella* isolates is higher than previously detected [98,99,100]. In the US, the resistance of *Salmonella* to Nal, Gen, and Cip has increased from 2.2%, 1.3%, and 0.3% in 2004 to 2.8%, 2.0%, and 0.5% in 2013, respectively [101]. Furthermore, the resistance of *Salmonella* isolates to Amp, Kan, Str, and Sul is higher in our study than previous reports from dairy feces in New York and other northeastern states of the US [92].

*E. coli* O157 isolates showed 73% MDR with higher resistance to aminoglycosides, macrolides, and folate antagonists (Table 3). These results were higher than previously detected from Colorado and southeastern US [102,103]. Similarly, the most detected MDR in *Salmonella* isolates (77%) was to aminoglycosides, macrolide, and folate antagonists (Table 3), which is higher than previously detected from Georgia and Washington [104,105]. The MDR resistance to Amp (penicillin)/Str (aminoglycosides)/Sul (folate antagonists) continued to increase from less than 1.5% in 1996 to 17% in 2010, 18.3% in 2011, 26.5% in 2012, and 45.5% in 2013 [106]. The rise in antimicrobial-resistant *Salmonella* might be attributed in part to the clonal spread of multidrug-resistance strains, differences in farming practices, or to the variations in antimicrobial use guidelines [107].

Interestingly, 100% of *L. monocytogenes* isolates recovered from poultry manure amended farms possessed resistance to Kan, Nal, and Lev, while 86%, 94.8%, and 89.6% of isolates recovered from dairy cattle manure amended farms possessed resistance to Kan, Nal, and Lev, respectively (Table S6). The resistance in *L. monocytogenes* isolates was significantly higher among tested antimicrobials than previously reported resistance in dairy farms in Tennessee [35]. Notably, 95.5% of *L. monocytogenes* isolates demonstrated resistance to nalidixic acid; however, *L. monocytogenes* has been previously reported to have intrinsic resistance to nalidixic acid [108].

*Campylobacter* isolates showed MDR (57.3%) to aminoglycosides, tetracycline, and penicillin (Table 3). This is higher than previous reports in North Carolina and southeastern US [103,109]. The resistance of *Campylobacter* isolates to Tet (63%), Cli (11.7%), Gen (25%), Kan (48.5%), and Str (22%) in this study is higher than the detected prevalence in the midwestern and northeastern US [110,111]. *Campylobacter* isolates recovered from poultry manure amended farms were not resistant to Tet and Cip (Table S7), however, resistance to these two antibiotics was higher in Ohio [82]. The resistance of *Campylobacter* isolates to Cip was lower than in the previous studies in the US, which increased from 12.8% in 2004 to 16.1% in 2012 [112]. Generally, the resistance trend of foodborne pathogens did not increase from 2016 to 2020 (Figure S1) compared to other studies [107,113,114,115,116]. Our study showed that antimicrobial resistance increased in dairy cattle more than in poultry manure amended farms regardless of the type of pathogen. However, other studies showed that there is increased antimicrobial resistance in both poultry and dairy cattle manure [24]. The observed trend might be due to improved animal management, change in the use of antimicrobials, the physicochemical property of the soil, or other environmental factors such as pH, temperature, oxygen, and the abundance of heavy metals [87,117,118].

In this study, *mphA*, *aadA*, *aphA1*, and *tet(A)* were the most frequently detected genes within *E. coli* O157 isolates and *blaTEM*, *tet(B)*, and *strA* were the most frequently detected genes within *Salmonella* isolates. Furthermore, *penA*, *ampC*, *lde*, *ermB*, *tet(O)*, and *aadB* were the most frequently detected genes from *L. monocytogenes* isolates, and *blaOXA-61*, *tet(O)*, and *aadE* were the most frequently detected genes in *Campylobacter* isolates (Figure S2). These results were different from the results obtained in previous studies [35,119,120,121]. The prevalence of *aadA* (75%) and *tet(A)* (50%) genes in *E. coli* O157 isolates [122] and *blaTEM* (66.6%) and *tet(B* 66.6%) genes in *Salmonella* isolates (Figure S2) were lower than the detected prevalence by McMillan et al. [123], whereas the detected prevalence of *penA* (50%), *ampC* (66.6%), and *ermB* (28%) genes in *L. monocytogenes* isolates is higher in our study than the detected prevalence of the same genes in fecal and environmental samples collected from Tennessee [35,124]. Further, the prevalence of *blaOXA-61* (90%) and *tet(O)* (79%) in *Campylobacter* isolates is higher than their prevalence in fecal samples collected from northwestern Ohio and Michigan [71,82]. Interestingly, *aadA*, *aph(6)-I*, *bla*, *tet*, *tet*(A), and *sulII* were the most prevalent ARGs found in different foodborne pathogens in Pennsylvania, Maryland, New York, New Mexico, Minnesota, and California [125]. However, in our study, *aadA* and *blaTEM* were more prevalent genes. Our results demonstrated that animal manure are important reservoirs of ARGs; thus, it is recommended to develop specific management practices such as aerobic and hyperthermophilic composting for farm amendment of different types of animal manure [126,127,128].

Resistance of *Salmonella* isolates to Azi cannot be explained by *ermB*; similarly, resistance of *L. monocytogen* isolates to Cefo, Azi, and Tri cannot be explained by the resistance genes *cfxA*, *mefA*, and *sulI.* Further, resistance of *Campylobacter* isolates to Gen cannot be explained by *aph-3-1*. The observed antimicrobial resistance could be due to different resistance mechanisms such as antibiotic modification and a multidrug efflux pump, which confers a broad spectrum of resistance [129]. Notably, specific alleles of aminoglycoside resistance have been detected in several studies of NARMS US food animal isolates [35,119,120,121,130]. Taken together, our data suggest that the *E. coli* O157, *Salmonella*, *L. monocytogenes* and *Campylobacter* occurring in dairy cattle and poultry farms have the genetic potential that is necessary for exhibiting resistance to antimicrobials. We found that there was a significant positive association between ARGs and the corresponding antimicrobials (Figure 3). However, the existence of significant correlations between ARGs and other unrelated antimicrobials belonging to different classes was also observed. This correlation could be due to the presence of multiple resistance genes on the same mobile genetic element, as many genes can play a role in the display of similar resistance phenotypes [131,132,133].

## 5. Conclusions

Livestock and poultry are the most important reservoirs for foodborne pathogens. They can transmit pathogenic bacteria to agricultural farms through manure amendments. Our results revealed that the prevalence and antimicrobial resistance of *E. coli* O157*, Salmonella*, *L. monocytogenes*, and *Campylobacter* varied among different farm types amended with animal manure in Northeastern Ohio. Dairy cattle manure amended farms were more frequently contaminated with the aforementioned pathogens and contained more resistant foodborne pathogens to the tested antimicrobials compared to poultry manure amended farms. Most of the bacterial isolates were resistant to multiple antimicrobials with genotypic diversity in ARGs. Therefore, there is a need to (1) track antibiotic use in different types of food animals, (2) restrict the use of antimicrobials in veterinary practices to reduce the antimicrobial resistant pathogens that might enter the agricultural farms through manure amendment and impact human health, and (3) control foodborne pathogens in agricultural farms to limit the transmission of these pathogens to humans. Further studies are needed to understand the impact of animal manure amendment in the food chain and in the environment.

## Figures and Tables

**Table 1:** Total number of samples collected between October 2016 and October 2020.

Year	No. of Farms	Total No of Samples	Farm Amendment Type		Sample Type	
			Dairy Manure	Poultry Manure	Manure	Soil
2016/2017	11	252	131	121	112	140
2018	11	262	142	120	120	142
2019	11	221	113	108	95	126
2020	10	109	55	54	52	57
Total		844	441	403	379	465

**Table 2:** Prevalence of foodborne pathogen from different sources.

Pathogen	No. of Isolates	Farm Amendment Type		Sample Type	
		Dairy Manure (*n* = 441) No. (%)	Poultry Manure (*n* = 403) No. (%)	Manure (*n* = 379) No. (%)	Soil (*n* = 465) No. (%)
*E. coli* O157	15	13 (2.9)	2 (0.5)	11 (2.9)	4 (0.9)
*Salmonella*	13	6 (1.4)	7 (1.7)	12 (3.2)	1 (0.2)
*L. monocytogenes*	67	58 (13.1)	9 (2.2)	47(12.4)	20 (4.3)
*Campylobacter*	68	66 (14.9)	2 (0.5)	67 (17.7)	1 (0.2)
Total positive	163	143 (87.7) *	20 (12.2)	137 (84) *	26 (15.9)

* Significantly higher prevalence of foodborne pathogens (*p* < 0.05).

**Table 3:** Antimicrobial resistance profile of the foodborne pathogens.

Antimicrobial Class	Antimicrobials	*E. coli* O157 (*n* = 15)			*Salmonella* (*n* = 13)			*L. monocytogenes* (*n* = 67)			*Campylobacter* (*n* = 68)		
		R No. (%)	I No. (%)	S No. (%)	R No. (%)	I No. (%)	S No. (%)	R No. (%)	I No. (%)	S No. (%)	R No. (%)	I No. (%)	S No. (%)
Penicillin	Amp	6 (40)	0	9 (60)	6 (46)	2 (15.3)	5 (38.4)	60 (89.5)	0	7 (10.5)	63 (97)	0	5 (3)
	Pen	NA	NA	NA	NA	NA	NA	32 (47.7)	0	35 (52.3)	68 (100)	0	0
Phenicol	Chl	0	0	15 (100)	1 (7.8)	0	12 (92.2)	42 (61.7)	8 (11.7)	17 (25)	6 (5.9)	0	62 (94.1)
	Flo	NA	NA	NA	NA	NA	NA	NA	NA	NA	2 (2.9)	0	66 (97)
Quinolones	Cip	0	5 (33.3)	10 (66.6)	1 (7.6)	7(46)	5 (38.4)	53 (79)	1 (1.4)	13 (19.6)	4 (5.8)	0	64 (94.2)
	Nal	0	0	15 (100)	7 (53.8)	1(7.8)	5 (38.4)	64 (95.5)	0	3 (4.5)	23 (33.8)	0	45 (66.2)
Aminoglycosides	Kan	9 (60)	1 (6.7)	5 (33.3)	6 (46)	2 (15.3)	5 (38.4)	59 (88)	0	8 (12)	33 (48.5)	4 (5.8)	31 (45.5)
	Gen	13 (86.6)	1 (6.7)	1 (6.7)	10 (76.9)	1 (7.8)	2 (15.3)	52 (77.6)	0	15 (22.3)	17 (25)	0	51 (75)
	Str	8 (54)	0	7 (46)	11 (84.6)	0	2 (15.3)	66 (98.5)	1 (1.5)	0	15 (22)	12 (17.6)	41 (60.2)
Tetracyclines	Tet	2 (23)	2 (13)	11 (73.3)	3 (23)	1 (7.8)	9 (69.2)	23 (34.3)	0	44 (65.6)	43 (63.2)	0	25 (36.8)
Macrolides	Ery	NA	NA	NA	NA	NA	NA	25 (37.3)	0	42 (62.7)	67 (98.5)	0	1 (1.5)
	Azi	11(73.3)	0	4 (26.6)	10 (77)	0	3 (23)	67 (100)	0	0	6 (5.9)	0	62 (94.1)
Cephems	Ceft	0	0	15 (100)	1 (7.8)	1 (7.8)	11 (84.6)	67 (100)	0	0	NA	NA	NA
	Cefo	1 (6.7)	0	14 (93.1)	1 (7.8)	1 (7.8)	11 (84.6)	67 (100)	0	0	NA	NA	NA
β–lactam	Amo	1 (6.7)	0	14 (93.1)	1 (7.8)	0	12 (92.2)	NA	NA	NA	NA	NA	NA
Folate pathway Antagonists	Tri	15 (100)	0	0	13 (100)	0	0	67 (100)	0	0	NA	NA	NA
	Sul	15 (100)	0	0	13 (100)	0	0	NA	NA	NA	NA	NA	NA
Polymyxins	Col	1 (6.7)	0	14 (93.1)	2 (15.3)	0	11 (84.6)	NA	NA	NA	NA	NA	NA
Penem	Mer	0	0	15 (100)	0	0	13 (100)	67 (100)	0	0	NA	NA	NA
Glycopeptide	Van	NA	NA	NA	NA	NA	NA	45 (67)	22 (33)	0	NA	NA	NA
Oxazolidinone	Lin	NA	NA	NA	NA	NA	NA	39 (58)	28 (42)	0	NA	NA	NA
Nitrofuran	Nit	NA	NA	NA	NA	NA	NA	6 (8.9)	59 (86)	2 (2.9)	NA	NA	NA
Lincomycin	Cli	NA	NA	NA	NA	NA	NA	67 (100)	0	0	8 (11.7)	0	60 (80.8)
Rifamycins	Rif	NA	NA	NA	NA	NA	NA	67 (100)	0	0	NA	NA	NA
Fluoroquinolone	Lev	NA	NA	NA	NA	NA	NA	61 (91)	6 (9)	0	NA	NA	NA
Ketolides	Tel	NA	NA	NA	NA	NA	NA	NA	NA	NA	4 (5.8)	0	64 (94.2)

S, susceptible; I, intermediate; R, resistance; NA, not applicable. The antimicrobial resistance was determined using the broth microdilution method [45]. Results are shown as number of isolates with the percentage given in parentheses. Amp: ampicillin, Chl: chloramphenicol, Cip: ciprofloxacin, Kan: kanamycin, Nal: nalidixic acid, Str: streptomycin, Tet: tetracycline, Gen: gentamycin, Sul: sulfisoxazole, Ceft: ceftriaxone, Amo: amoxicillin, Cefo: cefoxitin, Azi: azithromycin, Tri: trimethoprim sulfamethoxazole, Mer: meropenem, Col: colistin, Pen: penicillin G, Ery: erythromycin, Van: vancomycin, Lin: linezolid, Nit: nitrofurantoin, Cli: clindamycin, Rif: rifampicin, Lev: levofloxacin, Tel: telithromycin, Flo: florfenicol.

## Data Availability

Not applicable.

## Supplementary Materials

The following are available online at https://www.mdpi.com/article/10.3390/antibiotics10121450/s1, Figure S1: Antimicrobial resistance trend in different foodborne pathogens between 2016 and 2020; Figure S2: Antimicrobial resistance genes of; (A) *E. coli O157* (B) *Salmonella* (C) *L. monocytogenes* (D) *Campylobacter* recovered from different manure type; Table S1: PCR primer used for the identification of different foodborne pathogens; Table S2: Interpretive Criteria for Minimum Inhibitory Concentrations; Table S3: Antimicrobial resistance gene primers used for the detection of antimicrobial resistance genes in *E. coli* O157, *Salmonella L. monocytogenes* and *Campylobacter;* Table S4: Antimicrobial resistance profile of the *E. coli* O157 isolates according to manure amendment type and the source of the isolates; Table S5: Antimicrobial resistance profile of the *Salmonella* isolates according to manure amendment type and the source of the isolates; Table S6: Antimicrobial resistance profile of the *L. monocytogenes* isolates according to manure amendment type and the source of the isolates; Table S7: Antimicrobial resistance profile of the *Campylobacter* isolates according to manure amendment type and the source of the isolates.
