# Supplementary material for: Prevalence and Antimicrobial Resistance Profiles of Foodborne Pathogens Isolated from Dairy Cattle and Poultry Manure Amended Farms in Northeastern Ohio, the United States
Source: Antibiotics (Basel). 2021 Nov 25;10(12):1450. doi: 10.3390/antibiotics10121450 (PMC8698512; doi:10.3390/antibiotics10121450)
Supplement: Supplementary file 1 [file antibiotics-10-01450-s001.zip › antibiotics-1442524-supplementary.pdf]

Table S1. PCR primer used for the identification of different foodborne pathogens.

Pathogen	Target gene	Primer sequence	Amplicon size	PCR conditions	Reference
E. coli O157:H7	O157	RfbF: GTGTCCATTTATACGGACATCCATG<br>RfbR: CCTATAACGTCATGCCAATATTGCC	292	Initial denaturation step at 94°C for 3 min; 35 cycles of 94°C for 30s, 59°C for 60s, and 72°C for 60s; and one final cycle of 72°C for 7 min	(Hu et al., 1999)
	H7	FLICh7: FGCGCTGTCGAGTTCTATCGAGC<br>FLICh7-RCAACGGTGACTTATCGCCATTCC	625	Initial denaturation step at 94°C for 3 min; 35 cycles of 94°C for 30s, 59°C for 60s, and 72°C for 60s; and one final cycle of 72°C for 7 min	(Hu et al., 1999)
	Intimin	IntF : GACTGTCGATGCATCAGGCAAAG<br>IntR : TTGGAGTATTAACATTAACCCCAGG	368	Initial denaturation step at 94°C for 3 min; 35 cycles of 94°C for 30s, 59°C for 60s, and 72°C for 60s; and one final cycle of 72°C for 7 min	(Hu et al., 1999)
	SLT-I	SLT-IF: TGTAACTGGAAAGGTGGAGTATAC<br>SLT-IR: GCTATTCTGAGTCAACGAAAAATAAC	210	Initial denaturation step at 94°C for 3 min; 35 cycles of 94°C for 30s, 59°C for 60s, and 72°C for 60s; and one final cycle of 72°C for 7 min	(Hu et al., 1999)
	SLT-II	SLT-IIF: GTTTTTCTTCGGTATCCTATTCCG<br>SLT-IIR: GATGCATCTCTGGTCATTGTATTAC	483	Initial denaturation step at 94°C for 3 min; 35 cycles of 94°C for 30s, 59°C for 60s, and 72°C for 60s; and one final cycle of 72°C for 7 min	(Hu et al., 1999)
Salmonella	*invA*	S139F: GTGAAATTATCGCCACGTTCGGGCAA<br>S141R: TCATCGCACCGTCAAAGGAACC	284	Initial denaturation step at 94°C for 3 min; 35 cycles of 94°C for 1min, 53°C for 2min, and 72°C for min; and one final cycle of 72°C for 7 min	(Rahn et al., 1992)
Listeria monocytogenes	*Imo0737*	F: AGGGCTTCAAGGACTTACCC<br>R: ACGATTTCTGCTTGCCATTC	691	Initial denaturation step at 94°C for 3 min; 35 cycles of 94°C for 24s, 53°C for 69s, and 72°C for 69s; and one final cycle of 72°C for 7 min	(Doumith et al., 2004)
	Imo1118	F: AGGGGTCTTAAATCCTGGAA<br>R: CGGCTTGTTCGGCATACTTA	906	Initial denaturation step at 94°C for 3 min; 35 cycles of 94°C for 24s, 53°C for 69s, and 72°C for 69s; and one final cycle of 72°C for 7 min	(Doumith et al., 2004)
	ORF2819	F: AGCAAAATGCCAAAACTCGT<br>R: CATCACTAAAGCCTCCCATTG	471	Initial denaturation step at 94°C for 3 min; 35 cycles of 94°C for 24s, 53°C for 69s, and 72°C for 69s; and one final cycle of 72°C for 7 min	(Doumith et al., 2004)

	ORF2110	F: AGTGGACAATTGATTGGTGAA<br>R: CATCCATCCCTTACTTTGGAC	597	Initial denaturation step at 94°C for 3 min; 35 cycles of 94°C for 24s, 53°C for 69s, and 72°C for 69s; and one final cycle of 72°C for 7 min	(Doumith et al., 2004)
	hylA	F: CCTAACATATCCAGGTGCTCTC<br>R: CTGATTGCGCCGAAGTTTAC	350	Initial denaturation step at 94°C for 3 min; 35 cycles of 94°C for 24s, 53°C for 69s, and 72°C for 69s; and one final cycle of 72°C for 7 min	(Doumith et al., 2004)
Campylobacter spp.	16s rRNA	C412F: GGATGACACTTTTCGGAGC<br>C1228R: CATTGTAGCACGTGTGTC	816	Initial denaturation step at 94°C for 15 min; 25 cycles of 95°C for 30s, 58°C for 90s, and 72°C for 60s; and one final cycle of 72°C for 7 min	(Yamazaki-Matsune et al., 2007)

Table S2: Interpretive Criteria for Minimum Inhibitory Concentrations

Bacteria	Antimicrobial class	Mode of action	Antimicrobial Agents	Minimum Inhibitory Concertation interpretive criteria (µg/mL) *			Reference
				S	I	R	
E. coli O157	Aminoglycosides	Inhibit protein synthesis	Streptomycin	≤32	-	≥64	(NARMS, 2019)
			Kanamycin	≤16	32	≥64	(CLSI, 2015)
			Gentamycin	≤4	8	≥16	(CLSI, 2015)
	β–lactam	Inhibit cell wall synthesis	Amoxicillin	≤8	16	≥32	(NARMS, 2019)
	Quinolones	Inhibit the bacterial DNA gyrase enzyme, and replication	Nalidixic acid	≤16	-	≥32	(NARMS, 2019)
			Ciprofloxacin	≤1	2	≥4	(CLSI, 2015)
	Phenicols	Inhibit protein synthesis	Chloramphenicol	8	16	32	(CLSI, 2015)
	Tetracyclines	Inhibit protein synthesis	Tetracycline	≤4	8	≥16	(CLSI, 2015)
	Cephems	Inhibit cell wall synthesis	Ceftriaxone	≤1	2	≥4	(NARMS, 2019)
			Cefoxitin	≤8	16	≥32	(NARMS, 2019)
	Macrolides	Inhibit protein synthesis	Azithromycin	≤16	-	≥32	(NARMS, 2019)
	Folate pathway antagonists	Inhibit folate action	Sulfisoxazole	≤256	-	≥512	(NARMS, 2019)
			Trimethoprim sulfamethoxazole	≤2	-	≥4	(NARMS, 2019)
	Penicillin	Inhibit cell wall synthesis	Ampicillin	≤8	16	≥32	(CLSI, 2015)
	Penems	Inhibit cell wall synthesis	Meropenem	≤1	2	≥4	(NARMS, 2019)
	Polymyxins	Disrupt cell wall integrity	Colistin	≤2	-	≥2	(Matuschek et al., 2018)
Salmonella	Aminoglycosides	Inhibit protein synthesis	Streptomycin	≤32	-	≥64	(NARMS, 2019)
			Kanamycin	≤16	32	≥64	(CLSI, 2015)
			Gentamycin	≤4	8	≥16	(CLSI, 2015)
	β–lactam	Inhibit cell wall synthesis	Amoxicillin	≤8	16	≥32	(NARMS, 2019)
	Quinolones	Inhibit the bacterial DNA gyrase enzyme, and replication	Ciprofloxacin	≤0.06	0.12-0.5	≥1	(CLSI, 2015)
			Nalidixic acid	≤16	-	≥32	(NARMS, 2019)
	Phenicols	Inhibit protein synthesis	Chloramphenicol	≤8	16	≥32	(CLSI, 2015)
	Tetracyclines	Inhibit protein synthesis	Tetracycline	≤4	8	≥16	(CLSI, 2015)
	Cephems	Inhibit cell wall synthesis	Ceftriaxone	≤1	2	≥4	(NARMS, 2019)
			Cefoxitin	≤8	16	≥32	(NARMS, 2019)
	Macrolides	Inhibit protein synthesis	Azithromycin	≤16	-	≥32	(NARMS, 2019)

	Folate pathway antagonists	Inhibit folate action	Sulfisoxazole	≤256	-	≥512	(NARMS, 2019)
			Trimethoprim sulfamethoxazole	≤2	-	≥4	(NARMS, 2019)
	Penicillin	Inhibit cell wall synthesis	Ampicillin	≤8	16	≥32	(CLSI, 2015)
	Penem	Inhibit cell wall synthesis	Meropenem	≤1	2	≥4	(NARMS, 2019)
	Polymyxins	Disruption of cell wall integrity	Colistin	≤2	-	≥2	(Matuschek et al., 2018)
L. monocytogenes	Penicillin	Inhibit cell wall synthesis	Ampicillin	≤1	-	≥1	(EUCAST, 2018)
			Penicillin G	≤1	-	≥1	(EUCAST, 2018)
	Phenicol	Inhibit protein synthesis	Chloramphenicol	≤8	16	≥32	(Ruiz-Bolivar et al., 2011)
	Quinolones	Inhibit the bacterial DNA gyrase enzyme, and replication	Ciprofloxacin	≤1	2	≥4	(Ruiz-Bolivar et al., 2011)
			Nalidixic acid	≤8	-	≥16	(Ruiz-Bolivar et al., 2011)
	Aminoglycosides	Inhibit protein synthesis	Kanamycin	≤16	32	≥64	(CLSI, 2015)
			Gentamycin	≤4	8	≥16	(CLSI, 2015)
			Streptomycin	≤8	-	≥16	(CLSI, 2015)
	Macrolides	Inhibit protein synthesis	Erythromycin	≤0.5	1-4	≥8	(Ruiz-Bolivar et al., 2011)
			Azithromycin	≤0.06	-	≥1	(Madeo et al., 2015)
	Tetracyclines	Inhibit protein synthesis	Tetracycline	≤4	8	≥16	(Ruiz-Bolivar et al., 2011)
	Cephems	Inhibit cell wall synthesis	Ceftriaxone	≤4	-	≥8	((Madeo et al., 2015)
			Cefoxitin	≤4	-	≥8	(Chen et al., 2018)
	Lincomycin	Inhibit protein synthesis	Clindamycin	≤0.06	-	2	(Madeo et al., 2015)
	Folate pathway antagonists	Inhibit folate action	Trimethoprim sulfamethoxazole	≤0.06	-	≥0.06	(EUCAST, 2020)
	Nitrofuran	Inactivate ribosomal protein	Nitrofurantoin	≤32	64	≥128	(Chen et al., 2018)
	Glycopeptide	Inhibit cell wall synthesis	Vancomycin	≤0.125	-	2	((Granier et al., 2011)
	Fluoroquinolone	Inhibit DNA synthesis	Levofloxacin	≤0.06	-	2	(Madeo et al., 2015)
	Oxazolidinone	Inhibit protein synthesis	Linezolid	≤0.5	-	4	(Granier et al., 2011)
	Penem	Inhibit cell wall synthesis	Meropenem	≤0.25	-	≥0.25	(EUCAST, 2020)
	Antimycobacterial	Inhibition of RNA synthesis	Rifampicin	<0.25	-	0.5	(Granier et al., 2011)

Campylobacter	Penicillin	Inhibit cell wall synthesis	Ampicillin	≤8	16	≥32	(Kashoma et al., 2015)
			Penicillin	≤1	-	≥64	(Haruna et al., 2012)
	Phenicol	Inhibit protein synthesis	Chloramphenicol	≤16	-	≥32	(NARMS, 2019)
			Florfenicol	≤4		≥8	(NARMS, 2019)
	Quinolones	Inhibit the bacterial DNA gyrase enzyme, and replication	Ciprofloxacin	≤0.5	-	≥1	(NARMS, 2019)
			Nalidixic acid	≤16		≥32	(NARMS, 2019)
	Aminoglycosides	Inhibit protein synthesis	Streptomycin	≤2	4	≥8	(Kashoma et al., 2015)
			Gentamycin	≤2	-	≥4	(NARMS, 2019)
			Kanamycin	≤16	32	≥64	(CLSI, 2015)
	Macrolides	Inhibit protein synthesis	Erythromycin	≤4	-	≥8	(NARMS, 2019)
			Azithromycin	≤0.25		≥0.5	(NARMS, 2019)
	Tetracyclines	Inhibit protein synthesis	Tetracycline	≤1	-	≥2	(NARMS, 2019)
	ketolides	Inhibit protein synthesis	Telithromycin	≤4		≥8	(NARMS, 2019)
	Lincomycin	Inhibit protein synthesis	Clindamycin	≤0.5		≥1	(NARMS, 2019)

[*]S: Susceptible, I: Intermediate, R: Resistance

Table S3: Antimicrobial resistance gene primers used for the detection of antimicrobial resistance genes in *E. coli* O157, *Salmonella L. monocytogenes* and *Campylobacter*

**Foodborne pathogen**	**Antimicrobial agent**	**Resistant gene**	**Primer[a]: Nucleotide sequence**	**Resistance mechanism**	**Annealing Temp (°C)**	**Fragment length (bp)**	**References**
E. coli O157	Tetracycline	*tet(A)*	F: GCTACATCCTGCTTGCCTTC<br>R: CATAGATCGCCGTGAAGAGG	Efflux pump	50	210	(Bryan et al., 2004)
	Gentamicin	*aac(3)-IV*	F: CTTCAGGATGGCAAGTTGGT<br>R: TCATCTCGTTCTCCGCTCAT	Aminoglycoside acetyltransferase	50	286	(Saenz et al., 2004)
	Streptomycin	*aadA*	F: GCAGCGCAATGACATTCTTG<br>R: ATCCTTCGGCGCGATTTTG	Aminoglycoside adenyltransferase	50	282	(Saenz et al., 2004)
	Kanamycin	*aphA1*	F: ATGGGCTCGCGATAATGTC<br>R: CTCACCGAGGCAGTTCCAT	Aminoglycoside phosphoryltranferase	50	600	(Rosengren et al., 2009)
	Trimethoprim-sulfamethoxazole	*sulII*	F: CGGCATCGTCAACATAACCT<br>R: TGTGCGGATGAAGTCAGCTC	Target modification	52	721	(Srinivasan et al., 2005)
	Azithromycin	*mph(A)*	F: GTGAGGAGGAGCTTCGCGAG<br>R: TGCCGCAGGACTCGGAGGTC	Target modification	59	403	(Cho et al., 2019)
Salmonella	Ampicillin	*bla*$_{TEM-1}$	F: CAGCGGTAAGATCCTTGAGA<br>R: ACTCCCCGTCGTGTAGATAA	β – Lactamase	49	643	(Chen et al., 2004)
	Streptomycin	*strA*	F: CTTGGTGATAACGGCAATTC<br>R: CCAATCGCAGATAGAAGGC	Aminoglycoside phosphoryltranferase	47	548	(Gebreyes and Altier, 2002)
	Tetracycline	*tet(B)*	F: TTGGTTAGGGGCAAGTTTTG<br>R: GTAATGGGCCAATAACACCG	Efflux pump	48.5	659	(Chen et al., 2004)
	Gentamicin	*aac(3)-IVa*	F: GATGGGCCACCTGGACTGAT<br>R: GCGCTCACAGCAGTGGTCAT	Aminoglycoside acetyltransferase	54.5	462	(Chen et al., 2004)
	Trimethoprim sulfamethoxazole	*sulII*	F: CCTGTTTCGTCCGACACAGA<br>R: GAAGCGCAGCCGCAATTCAT	Target modification	55	435	(Chen et al., 2004)
	Azithromycin	*ermB*	F: GAAAAAGTACTCAACCAAATA	Target modification	45	639	(Mąka et al., 2015)

			R: AATTTAAGTACCGTTACT				
L. monocytogen	Ampicillin	*ampC*	F: TTCTATCAAMACTGGCARC<br>R: CCYTTTTATGTACCCAYGA	β – Lactamase	45	550	(Srinivasan et al., 2005)
	Ciprofloxacin	*Lde*	F: ATCGTGAACTTAATGGTGG<br>R: ATCCTCATATAACTCAAGCG	Efflux pump	45	1518	(Godreuil et al., 2003)
	Erythromycin	*ermB*	F: GAAAAGGTACTCAACCAAATA<br>R: AGTAACGGTACTTAAATTGTTTAC	Target modification	46	639	(Srinivasan et al., 2005)
	Tetracycline	*Tet(O)*	F: AATGAAGATTCCGACAATTTR<br>R: CTCATGCGTTGTAGTATTCCA	Efflux pump	45	781	(Li et al., 2007)
	Gentamicin	*aadB*	F: GAGCGAAATCTGCCGCTCTTG<br>R: CTGTTACAACGGACTGGCCGC	Aminoglycoside adenyltransferase	54	310	(Srinivasan et al., 2005)
	Penicillin G	*penA*	F: ATCGAACAGGCGACGATGTC<br>R: GATTAAGACGGTGTTTTACGG	Transpeptidases	46	500	(Srinivasan et al., 2005)
	Cefoxitin	*cfxA*	F: GCTCAAACAGATAGTTTTAT<br>R: GGCAACATTGTGAGCTG	β – Lactamase	50	333	(Maung et al., 2019)
	Clindamycin	*mefA*	F: AGTATCATTAATCACTAGTGC<br>R: TTCTTCTGGTACTAAAAGTGG	Target modification	50	345	(Maung et al., 2019)
	Trimethoprim-sulfamethazole	*sulI*	F: CGGCGTGGGCTACCTGAACG<br>R: GCCGATCGCGTGAAGTTCCG	Target modification	50	433	(Iwu and Okoh, 2019)
Campylobacter	Ampicillin	*blaOXA-61*	F: AGAGTATAATACAAGCG<br>R: TAGTGAGTTGTCAAGCC	β - Lactamase	45	372	(Obeng et al., 2012)
	Streptomycin	*aadE*	F: GAACAGGATGAACGTATTCG<br>R: GCATATGTGCTATCCAGG	Aminoglycoside phosphoryltranferase	45	837	(Obeng et al., 2012)
	Tetracycline	*tet(O)*	F: GCGTTTTGTTTATGTGCG<br>R: ATGGACAACCCGACAGAAG	Antibiotic target protection	49	559	(Obeng et al., 2012)
	Gentamycin	*aph-3-1*	F: TGCGTAAAAGATACGGAAG<br>R: CAATCAGGCTTGATCCCC	Aminoglycoside adenyltransferase	49	701	(Obeng et al., 2012)

[a] F, forward primer; R, reverse primer

Table S4: Antimicrobial resistance profile of the *E. coli* O157 isolates according to manure amendment type and the source of the isolates**.**

Antimicrobial agents	Source of isolate and number of resistant isolates (%)											
	**Dairy cattle (n=13)**			**Poultry (n=2)**			**Manure (n= 11)**			**Soil (n=4)**		
	**R No. (%)**	**I No. (%)**	**S No. (%)**	**R No. (%)**	**I No. (%)**	**S No. (%)**	**R No. (%)**	**I No. (%)**	**S No. (%)**	**R No. (%)**	**I No. (%)**	**S No. (%)**
Amp	4 (30.7)	0	9 (69.3)	2 (100)	0	0	4 (36.3)	0	7 (63.6)	2 (50)	0	2 (50)
Chl	0	0	13 (100)	0	0	2 (100)	0	0	11 (100)	0	0	4 (100)
Cip	0	4 (30.7)	9 (69.3)	0	1 (50)	1 (50)	0	4(36.3)	7 (63.6)	0	1 (25)	3 (75)
Kan	8 (61.5)	0	5 (39.5)	1 (50)	1 (50)	0	7(63.3)	0	4(36.3)	2 (50)	1 (25)	1 (25)
Nal	0	0	13 (100)	0	0	2 (100)	0	0	11 (100)	0	0	4 (100)
Str	6 (46.2)	0	7 (53.8)	2 (100)	0	0	6(54.5)	0	5 (45.4)	2 (50)	0	2 (50)
Tet	1 (7)	1 (7)	11 (84.6)	1 (50)	1 (50)	0	1 (9)	1 (9)	9 (82)	1 (25)	1 (25)	2 (50)
Gen	11 (84.6)	1 (7.7)	1 (7.7)	2 (100)	0	0	10(91)	1 (9)	0	3 (75)	0	1 (25)
Amo	1 (7.7)	0	12 (82.3)	0	0	2 (100)	1(9)	0	10 (91)	0	0	4 (100)
Cefo	1 (7.7)	0	12 (82.3)	0	0	2 (100)	1(9)	0	10 (91)	0	0	4 (100)
Ceft	0	0	13 (100)	0	0	2 (100)	0	0	11 (100)	0	0	4(100)
Sul	13 (100)	0	0	2 (100)	0	0	11(100)	0	0	4 (100)	0	0
Tri	13 (100)	0	0	2 (100)	0	0	11(100)	0	0	4 (100)	0	0
Azi	11 (84.6)	0	2 (14)	0	0	2 (100)	9(81.8)	0	2 (18.2)	2 (50)	0	2 (50)
Mer	0	0	13 (100)	0	0	2 (100)	0	0	11 (100)	0	0	4 (100)
Col	1 (7.7)	0	12 (82.3)	0	0	2 (100)	0	0	11 (100)	1 (25)	0	3 (75)

S, susceptible; I, intermediate; R, resistance

The antimicrobial resistance was determined using the broth microdilution method. Results are shown as number of isolates with the percentage given in parentheses. Amp; ampicillin, Cip; ciprofloxacin, Gen; gentamicin, Str; streptomycin, Tet; tetracycline, Nal; nalidixic acid, Kan; kanamycin, Chl; chloramphenicol, Amo; amoxicillin, Cefo; cefoxitin, Ceft; ceftriaxone, Sul; sulfisoxazole, Tri; Trimethoprim, Azi; azithromycin, Mer: Meropenem, Col: Colistin

Table S5: Antimicrobial resistance profile of the *Salmonella* isolates according to manure amendment type and the source of the isolates**.**

**Antimicrobial agents**	**Source of isolate and number of resistant isolates (%)**											
	**Dairy cattle (n=6)**			**Poultry (n=7)**			**Manure (n= 12)**			**Soil (n=1)**		
	**R No. (%)**	**I No. (%)**	**S No. (%)**	**R No. (%)**	**I No. (%)**	**S No. (%)**	**R No. (%)**	**I No. (%)**	**S No. (%)**	**R No. (%)**	**I No. (%)**	**S No. (%)**
Amp	5 (83.3)	0	1 (16.7)	1 (14.2)	2 (28)	4 (57)	6 (50)	2 (17.3)	4 (33.3)	0	0	1 (100)
Chl	1 (16.7)	0	5 (83.3)	0	0	7 (100)	1 (8.3)	0	11 (91.7)	0	0	1 (100)
Cip	0	4 (66.6)	2 (33.3)	3 (42.8)	1 (14.2)	3 (42.8)	1 (8.3)	7 (58.3)	4 (33.3)	0	0	1 (100)
Kan	2 (33.3)	2 (33.3)	2 (33.3)	4 (57)	0	3 (43)	6 (50)	2 (17.3)	4 (33.3)	0	0	1 (100)
Nal	2 (33.3)	0	4 (66.6)	5 (71.4)	1 (14.2)	1 (14.2)	7 (58.3)	1(8.3)	4 (33.3)	0	0	1 (100)
Str	5 (83.3)	0	1 (16.7)	6 (85.7)	0	1 (14.2)	10 (83.3)	0	2 (16.7)	1 (100)	0	0
Tet	1 (16.7)	1 (16.7)	4 (66.6)	2 (28.5)	0	5 (71.4)	3 (25)	2 (16.6)	7 (58.3)	0	0	1 (100)
Gen	3 (50)	1 (16.7)	2 (33.3)	7 (100)	0	0	9 (75)	1 (8.3)	2 (17.3)	1(100)	0	0
Amo	1 (16.7)	0	5 (83.3)	0	0	7 (100)	1 (8.3)	0	11 (91.7)	0	0	1 (100)
Cefo	1 (16.7)	1 (16.7)	4 (66.6)	0	0	7 (100)	1 (8.3)	1 (8.3)	10 (83.3)	0	0	1 (100)
Ceft	1 (16.7)	0	5 (83.3)	0	0	7 (100)	1 (8.3)	0	11 (91.7)	0	0	1 (100)
Sul	6 (100)	0	0	7 (100)	0	0	12 (100)	0	0	1 (100)	0	0
Tri	6 (100)	0	0	7 (100)	0	0	12 (100)	0	0	1 (100)	0	0
Azi	3 (50)	0	3 (50)	7 (100)	0	0	9 (75)	0	3 (25)	1 (100)	0	0
Mer	0	0	6 (100)	0	0	7 (100)	0	0	12 (100)	0	0	1 (100)
Col	0	0	6 (100)	2 (28.5)	0	5 (71.5)	1 (8.3)	0	11 (91.7)	1 (100)	0	0

S, susceptible; I, intermediate; R, resistance

The antimicrobial resistance was determined using the broth microdilution method. Results are shown as number of isolates with the percentage given in parentheses. Amp; ampicillin, Cip; ciprofloxacin, Gen; gentamicin, Str; streptomycin, Tet; tetracycline, Nal; nalidixic acid, Kan; kanamycin, Chl; chloramphenicol, Amo; amoxicillin, Cefo; cefoxitin, Ceft; ceftriaxone, Sul; sulfisoxazole, Tri; Trimethoprim, Azi; azithromycin, Mer: Meropenem, Col: Colistin

Table S6: Antimicrobial resistance profile of the *L. monocytogens* isolates according to manure amendment type and the source of the isolates**.**

Antimicrobial agents	Source of isolate and number of resistant isolates (%)											
	**Dairy cattle (n=58)**			**Poultry (n=9)**			**Manure (n= 47)**			**Soil (n=20)**		
	**R No. (%)**	**I No. (%)**	**S No. (%)**	**R No. (%)**	**I No. (%)**	**S No. (%)**	**R No. (%)**	**I No. (%)**	**S No. (%)**	**R No. (%)**	**I No. (%)**	**S No. (%)**
Amp	52 (89.6)	0	6 (8.9)	8 (88.8)	0	1 (11.1)	42(89.3)	0	5 (10.7)	18 (90)	0	2 (10)
Chl	15 (25.6)	7 (12)	36 (62)	2 (22.2)	1 (11.1)	6 (66.6)	14(29.7)	6 (12.7)	27 (57.4)	3 (15)	2 (10)	15 (75)
Cip	46 (79.2)	1 (1.7)	11 (16.4)	7 (77.7)	0	2 (22.2)	38(80.8)	1 (2.1)	8 (17)	15 (75)	0	5 (25)
Kan	50 (86)	0	8 (84)	9 (100)	0	0	42(89.3)	0	5 (10.7)	17 (85)	0	3 (15)
Nal	55 (94.8)	0	3 (95.2)	9 (100)	0	0	44(93.6)	0	3 (6.3)	20 (100)	0	0
Str	58 (100)	0	0	8 (88.8)	0	1 (11.1)	47(100)	0	0	19 (95)	0	1 (10)
Tet	20 (34)	0	38(66)	3 (33.3)	0	6 (66.6)	15(31.9)	0	32 (68)	8 (40)	0	12 (60)
Gen	44 (75.8)	0	14 (24.2)	8 (88.8)	0	1 (11.1)	36(76.6)	0	11 (23.3)	16 (80)	0	4 (20)
Pen	28 (48.3)	0	30 (51.7)	4 (44.4)	0	5 (55.5)	24(51)	0	23 (49)	8 (40)	0	12 (60)
Ery	22 (37.9)	0	36 (62.1)	3 (33.3)	0	6 (66.6)	24(51)	0	23 (49)	1 (5)	0	19 (95)
Ceft	58 (100)	0	0	9 (100)	0	0	47(100)	0	0	20 (100)	0	0
Cli	58 (100)	0	0	9 (100)	0	0	47(100)	0	0	20 (100)	0	0
Lev	52(89.6)	6 (8.9)	0	9 (100)	0	0	42(89.3)	5 (10.7)	0	19 (95)	1 (5)	0
Rif	58 (100)	0	0	9 (100)	0	0	47(100)	0	0	20 (100)	0	0
Tri	58 (100)	0	0	9 (100)	0	0	47(100)	0	0	20 (100)	0	0
Van	39(67)	19(33)	0	6 (66.6)	3 (33.3)	0	31(65.9)	16 (34)	0	14 (70)	6 (30)	0
Mer	58(100)	0	0	9 (100)	0	0	47(100)	0	0	20 (100)	0	0
Lin	36(62)	22 (38)	0	3 (33.3)	6 (66.6)	0	29(62)	18 (38)	0	10 (50)	10 (50)	0
Nit	6 (8.9)	50 (86)	2 (3.6)	0	9 (100)	0	4(8.5)	41 (87)	2 (4.2)	2 (10)	18 (90)	0
Azi	58 (100)	0	0	9 (100)	0	0	47(100)	0	0	20 (100)	0	0
Cefo	58 (100)	0	0	9 (100)	0	0	47(100)	0	0	20 (100)	0	0

S, susceptible; I, intermediate; R, resistance

The antimicrobial resistance was determined using the broth microdilution method. Results are shown as number of isolates with the percentage given in parentheses. Amp; ampicillin, Cip; ciprofloxacin, Gen; gentamicin, Str; streptomycin, Tet; tetracycline, Nal; nalidixic acid, Kan; kanamycin, Chl; chloramphenicol, Cefo: Cefoxitin, Azi: Azithromycin, Nit: Nitrofurantoin, Lin: Linezolid, Mer: Meropenem, Van: Vancomycin, Tri: Trimethoprim, Rif: Rifampicin, Lev: Levofloxacin, Cli: Clindamycin, Ceft: Ceftriaxone, Ery: Erythromycin, Pen: Penicillin G

Table S7: Antimicrobial resistance profile of the *Campylobacter* isolates according to manure amendment type and the source of the isolates**.**

**Antimicrobial agents**	**Source of isolate and number of resistant isolates (%)**											
	**Dairy cattle (n=66)**			**Poultry (n=2)**			**Manure (n= 67)**			**Soil (n=1)**		
	**R No. (%)**	**I No. (%)**	**S No. (%)**	**R No. (%)**	**I No. (%)**	**S No. (%)**	**R No. (%)**	**I No. (%)**	**S No. (%)**	**R No. (%)**	**I No. (%)**	**S No. (%)**
Amp	61(92.4)	0	5 (7.6)	2 (100)	0	0	63 (94)	0	4 (6)	0	0	1 (100)
Chl	6(9)	0	60 (91)	0	0	2 (100)	6(8.9)	0	61 (91.1)	0	0	1 (100)
Cip	62(93.9)	0	4 (6.1)	0	0	2 (100)	62(92.5)	0	5(7.5)	0	0	1 (100)
Ery	66(100)	0	0	1 (50)	0	1(50)	67(100)	0	0	0	0	1 (100)
Kan	32 (48.4)	3	31 (46.9)	1 (50)	0	1(50)	32(47.7)	3 (4.4)	32 (47.7)	1 (100)	0	0
Nal	21(31.8)	0	45 (68.1)	2 (100)	0	0	23(34.3)	0	44 (65.7)	0	0	1 (100)
Str	15(22.7)	12 (18.1)	39 (59)	0	0	2 (100)	15(22.2)	12 (19.7)	40 (59.7)	0	0	1 (100)
Tet	43(65)	0	23 (35)	0	0	2 (100)	43(64)	0	24 (36)	0	0	1 (100)
Gen	16(24)	0	50 (76)	1 (50)	0	1 (50)	17(25.3)	0	50 (74.6)	0	0	1 (100)
Pen	66(100)	0	0	2 (100)	0	0	67(100)	0	0	1(100)	0	0
Tel	4(6)	0	62 (94)	0	0	2 (100)	4(5.9)	0	63 (94.1)	0	0	1 (100)
Cli	58(87.8)	0	8 (12.2)	0	0	2 (100)	58 (86.5)	0	9(13.4)	0	0	1 (100)
Azi	6(9)	0	60 (91)	0	0	2 (100)	6(8.9)	0	61 (91.1)	0	0	1 (100)
Flo	2(3)	0	64 (97)	0	0	2 (100)	2(2.9)	0	65 (97.1)	0	0	1 (100)

S, susceptible; I, intermediate; R, resistance

The antimicrobial resistance was determined using the broth microdilution method. Results are shown as number of isolates with the percentage given in parentheses. Amp; ampicillin, Cip; ciprofloxacin, Gen; gentamicin, Str; streptomycin, Tet; tetracycline, Nal; nalidixic acid, Kan; kanamycin, Chl; chloramphenicol, Pen: Penicillin G, Ery: Erythromycin, Tel: Telithromycin, Cli: Clindamycin, Azi: Azithromycin, Flo: Florfenicol

Bryan, A., Shapir, N., Sadowsky, M.J., 2004. Frequency and distribution of tetracycline resistance genes in genetically diverse, nonselected, and nonclinical Escherichia coli strains isolated from diverse human and animal sources. Applied and environmental microbiology 70, 2503-2507.

Chen, M., Cheng, J., Wu, Q., Zhang, J., Chen, Y., Xue, L., Lei, T., Zeng, H., Wu, S., Ye, Q., Bai, J., Wang, J., 2018. Occurrence, Antibiotic Resistance, and Population Diversity of Listeria monocytogenes Isolated From Fresh Aquatic Products in China. Front Microbiol 9, 2215.

Chen, S., Zhao, S., White, D.G., Schroeder, C.M., Lu, R., Yang, H., McDermott, P.F., Ayers, S., Meng, J., 2004. Characterization of multiple-antimicrobial-resistant salmonella serovars isolated from retail meats. Applied and environmental microbiology 70, 1-7.

Cho, S., Nguyen, H.A.T., McDonald, J.M., Woodley, T.A., Hiott, L.M., Barrett, J.B., Jackson, C.R., Frye, J.G., 2019. Genetic Characterization of Antimicrobial-Resistant Escherichia coli Isolated from a Mixed-Use Watershed in Northeast Georgia, USA. International journal of environmental research and public health 16, 3761.

CLSI 2015. Performance Standards for Antimicrobial Susceptibility Testing; Twenty-Fifth Informational Supplement. http://www.facm.ucl.ac.be/intranet/CLSI/CLSI-2015-M100-S25-original.pdf.

Doumith, M., Buchrieser, C., Glaser, P., Jacquet, C., Martin, P., 2004. Differentiation of the major Listeria monocytogenes serovars by multiplex PCR. Journal of clinical microbiology 42, 3819-3822.

EUCAST, 2018. European Committee on Antimicrobial Susceptibility Testing. http://www.eucast.org/fileadmin/src/media/PDFs/EUCAST_files/Breakpoint_tables/v_8.1_Breakpoint_Tables.pdf.

EUCAST 2020. EUCAST breakpoint tables.

Gebreyes, W.A., Altier, C., 2002. Molecular characterization of multidrug-resistant Salmonella enterica subsp. enterica serovar Typhimurium isolates from swine. Journal of clinical microbiology 40, 2813-2822.

Godreuil, S., Galimand, M., Gerbaud, G., Jacquet, C., Courvalin, P., 2003. Efflux pump Lde is associated with fluoroquinolone resistance in Listeria monocytogenes. Antimicrobial agents and chemotherapy 47, 704-708.

Granier, S.A., Moubareck, C., Colaneri, C., Lemire, A., Roussel, S., Dao, T.-T., Courvalin, P., Brisabois, A., 2011. Antimicrobial Resistance of Listeria monocytogenes Isolates from Food and the Environment in France over a 10-Year Period. Applied and Environmental Microbiology 77, 2788.

Haruna, M., Sasaki, Y., Murakami, M., Ikeda, A., Kusukawa, M., Tsujiyama, Y., Ito, K., Asai, T., Yamada, Y., 2012. Prevalence and Antimicrobial Susceptibility of Campylobacter in Broiler Flocks in Japan. Zoonoses and Public Health 59, 241-245.

Hu, Y., Zhang, Q., Meitzler, J.C., 1999. Rapid and sensitive detection of Escherichia coli O157:H7 in bovine faeces by a multiplex PCR. Journal of applied microbiology 87, 867-876.

Iwu, C.D., Okoh, A.I., 2019. Preharvest Transmission Routes of Fresh Produce Associated Bacterial Pathogens with Outbreak Potentials: A Review. International journal of environmental research and public health 16, 4407.

Kashoma, I.P., Kassem, I.I., Kumar, A., Kessy, B.M., Gebreyes, W., Kazwala, R.R., Rajashekara, G., 2015. Antimicrobial Resistance and Genotypic Diversity of Campylobacter Isolated from Pigs, Dairy, and Beef Cattle in Tanzania. Frontiers in microbiology 6, 1240-1240.

Li, Q., Sherwood, J.S., Logue, C.M., 2007. Antimicrobial resistance of Listeria spp. recovered from processed bison. Letters in applied microbiology 44, 86-91.

Madeo, M., Musumeci, R., Careddu, A.M.L., Amato, E., Pontello, M.M., Cocuzza, C.E., 2015. Antimicrobial susceptibility of Listeria monocytogenes isolates from human cases in northern Italy, 2008–2010: MIC determination according to EUCAST broth microdilution method. Journal of Chemotherapy 27, 201-206.

Matuschek, E., Åhman, J., Webster, C., Kahlmeter, G., 2018. Antimicrobial susceptibility testing of colistin – evaluation of seven commercial MIC products against standard broth microdilution for Escherichia coli, Klebsiella pneumoniae, Pseudomonas aeruginosa, and Acinetobacter spp. Clinical Microbiology and Infection 24, 865-870.

Maung, A.T., Mohammadi, T.N., Nakashima, S., Liu, P., Masuda, Y., Honjoh, K.I., Miyamoto, T., 2019. Antimicrobial resistance profiles of Listeria monocytogenes isolated from chicken meat in Fukuoka, Japan. Int J Food Microbiol 304, 49-57.

Mąka, Ł., Maćkiw, E., Ścieżyńska, H., Modzelewska, M., Popowska, M., 2015. Resistance to Sulfonamides and Dissemination of sul Genes Among Salmonella spp. Isolated from Food in Poland. Foodborne Pathogens and Disease 12, 383-389.

NARMS 2019. National Antimicrobial Resistance

Monitoring System for Enteric Bacteria, .

Obeng, A.S., Rickard, H., Sexton, M., Pang, Y., Peng, H., Barton, M., 2012. Antimicrobial susceptibilities and resistance genes in Campylobacter strains isolated from poultry and pigs in Australia. Journal of Applied Microbiology 113, 294-307.

Rahn, K., De Grandis, S.A., Clarke, R.C., McEwen, S.A., Galán, J.E., Ginocchio, C., Curtiss, R., 3rd, Gyles, C.L., 1992. Amplification of an invA gene sequence of Salmonella typhimurium by polymerase chain reaction as a specific method of detection of Salmonella. Mol Cell Probes 6, 271-279.

Rosengren, L.B., Waldner, C.L., Reid-Smith, R.J., 2009. Associations between antimicrobial resistance phenotypes, antimicrobial resistance genes, and virulence genes of fecal Escherichia coli isolates from healthy grow-finish pigs. Applied and environmental microbiology 75, 1373-1380.

Ruiz-Bolivar, Z., Neuque-Rico, M.C., Poutou-Pinales, R.A., Carrascal-Camacho, A.K., Mattar, S., 2011. Antimicrobial susceptibility of Listeria monocytogenes food isolates from different cities in Colombia. Foodborne pathogens and disease 8, 913-919.

Saenz, Y., Brinas, L., Dominguez, E., Ruiz, J., Zarazaga, M., Vila, J., Torres, C., 2004. Mechanisms of resistance in multiple-antibiotic-resistant Escherichia coli strains of human, animal, and food origins. Antimicrobial agents and chemotherapy 48, 3996-4001.

Srinivasan, V., Nam, H.M., Nguyen, L.T., Tamilselvam, B., Murinda, S.E., Oliver, S.P., 2005. Prevalence of antimicrobial resistance genes in Listeria monocytogenes isolated from dairy farms. Foodborne pathogens and disease 2, 201-211.

Yamazaki-Matsune, W., Taguchi, M., Seto, K., Kawahara, R., Kawatsu, K., Kumeda, Y., Kitazato, M., Nukina, M., Misawa, N., Tsukamoto, T., 2007. Development of a multiplex PCR assay for identification of Campylobacter coli, Campylobacter fetus, Campylobacter hyointestinalis subsp. hyointestinalis, Campylobacter jejuni, Campylobacter lari and Campylobacter upsaliensis. Journal of medical microbiology 56, 1467-1473.

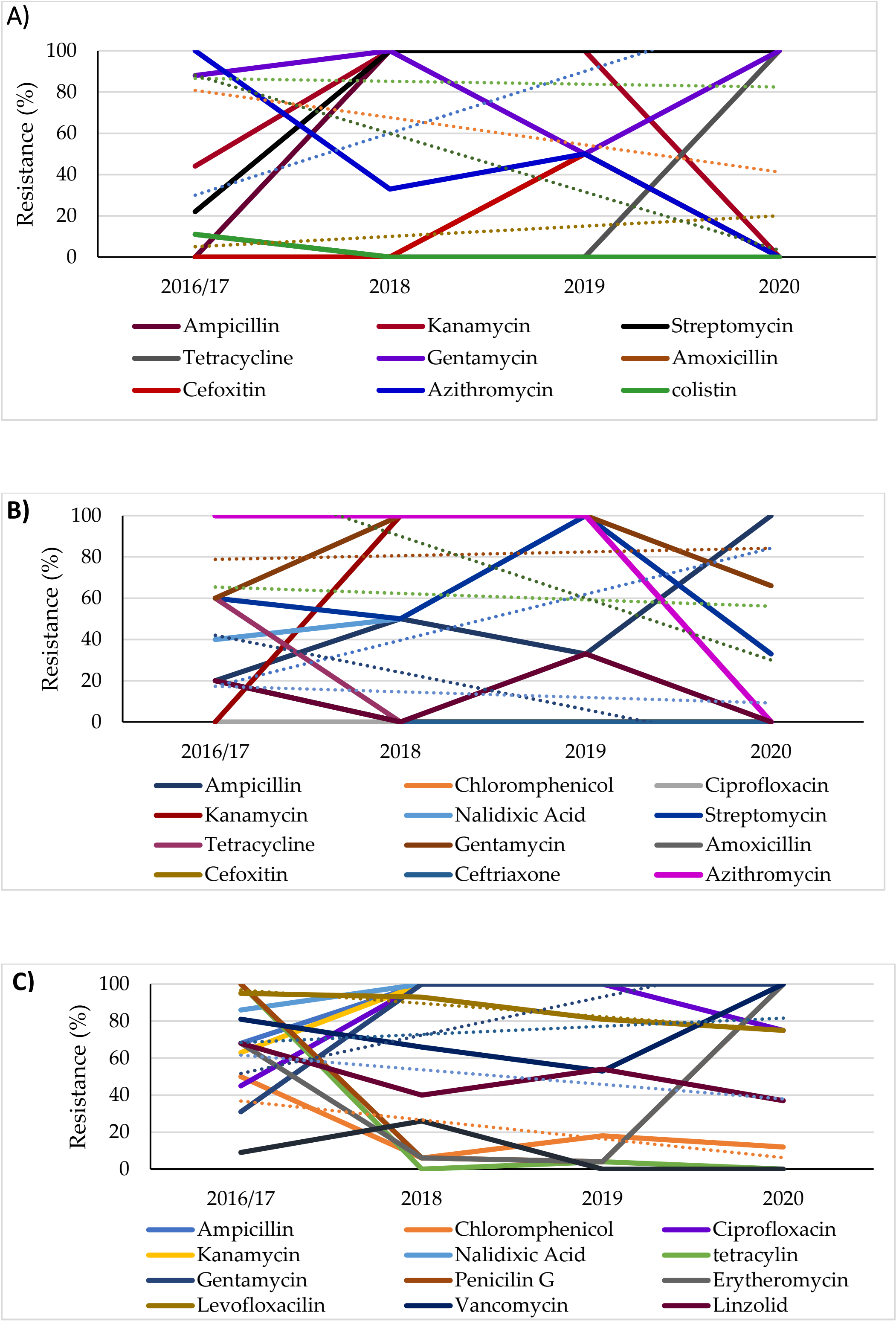
A)
Resistance (%)
100
80
60
40
20
0
2016/17
2018
2019
2020
Ampicillin
Kanamycin
Streptomycin
Tetracycline
Gentamycin
Amoxicillin
Cefoxitin
Azithromycin
colistin
B)
Resistance (%)
100
80
60
40
20
0
2016/17
2018
2019
2020
Ampicillin
Chloromphenicol
Ciprofloxacin
Kanamycin
Nalidixic Acid
Streptomycin
Tetracycline
Gentamycin
Amoxicillin
Cefoxitin
Ceftriaxone
Azithromycin
C)
Resistance (%)
100
80
60
40
20
0
2016/17
2018
2019
2020
Ampicillin
Chloromphenicol
Ciprofloxacin
Kanamycin
Nalidixic Acid
tetracylin
Gentamycin
Penicilin G
Erytheromycin
Levofloxacilin
Vancomycin
Linzolid

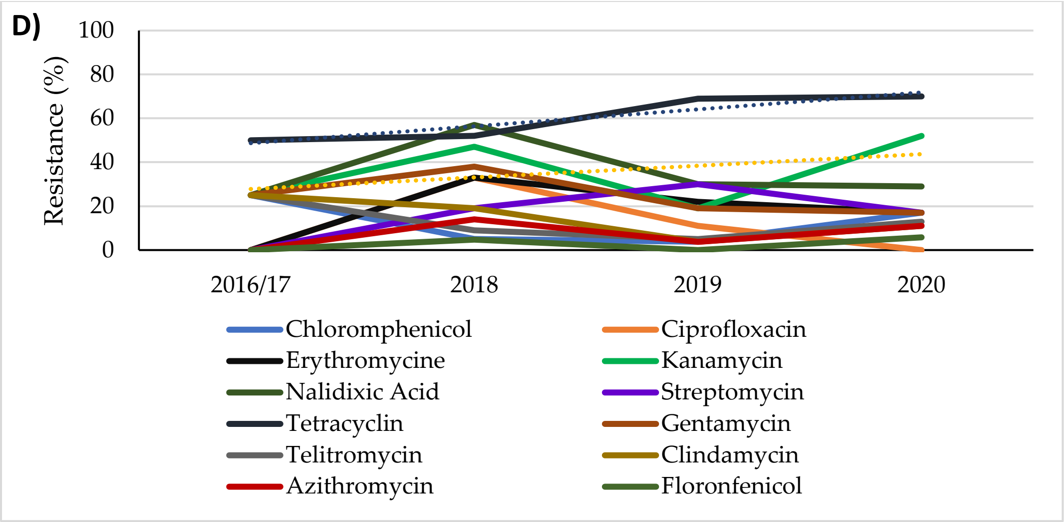

Figure S1. Antimicrobial resistance trend in different foodborne pathogens between 2016 and 2020.

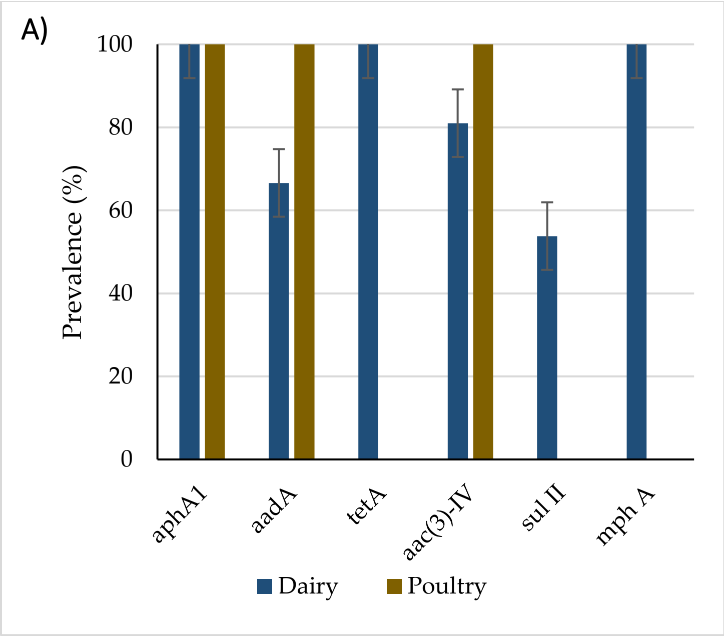
A)
100
80
60
40
20
0
Prevalence (%)
aphA1
aadA
tetA
aac(3)-IV
sul II
mph A
Dairy
Poultry

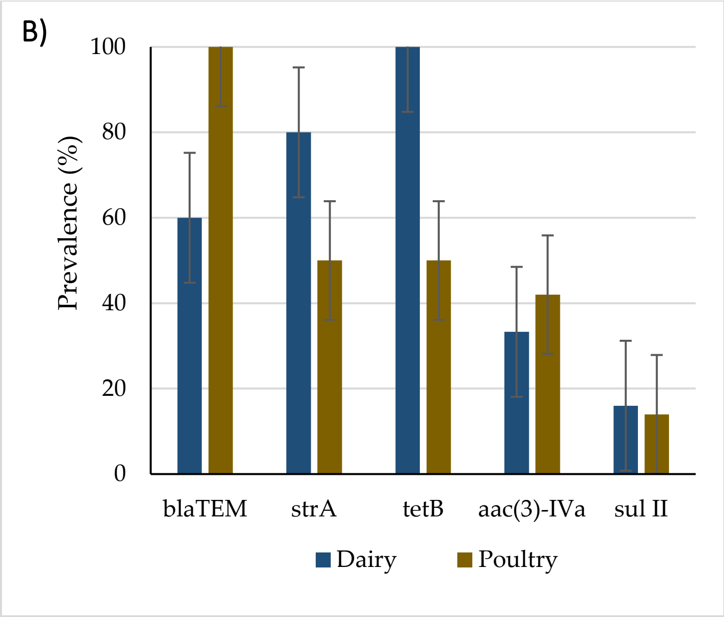
B)
100
80
60
40
20
0
Prevalence (%)
blaTEM
strA
tetB
aac(3)-IVa
sul II
Dairy
Poultry

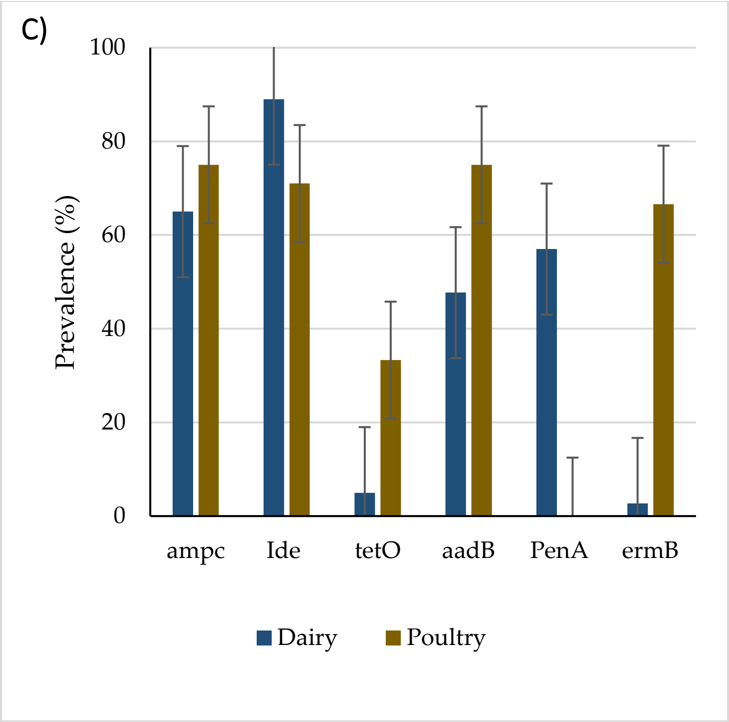

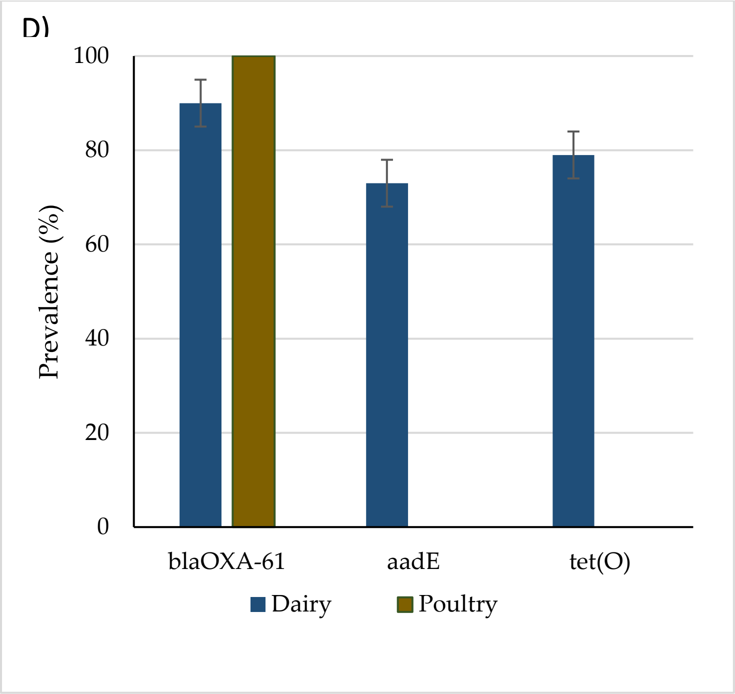

Figure S2. Antimicrobial resistance genes of; **A)** *E. coli O157* **B)** *Salmonella* **C)** *L. monocytogenes* **D)** *Campylobacter* recovered from different manure type.
